# Variants in *PPP2R2B* and *IGF2BP3* are associated with higher tau deposition

**DOI:** 10.1093/braincomms/fcaa159

**Published:** 2020-09-26

**Authors:** Vijay K Ramanan, Xuewei Wang, Scott A Przybelski, Sheelakumari Raghavan, Michael G Heckman, Anthony Batzler, Matthew L Kosel, Timothy J Hohman, David S Knopman, Jonathan Graff-Radford, Val J Lowe, Michelle M Mielke, Clifford R Jack, Ronald C Petersen, Owen A Ross, Prashanthi Vemuri

**Affiliations:** Department of Neurology, Mayo Clinic-Minnesota, Rochester, MN 55905, USA; Department of Health Sciences Research, Mayo Clinic-Minnesota, Rochester, MN 55905, USA; Department of Health Sciences Research, Mayo Clinic-Minnesota, Rochester, MN 55905, USA; Department of Radiology, Mayo Clinic-Minnesota, Rochester, MN 55905, USA; Division of Biomedical Statistics and Informatics, Mayo Clinic-Florida, Jacksonville, FL 32224, USA; Department of Health Sciences Research, Mayo Clinic-Minnesota, Rochester, MN 55905, USA; Department of Health Sciences Research, Mayo Clinic-Minnesota, Rochester, MN 55905, USA; Department of Neurology, Vanderbilt University Medical Center, Nashville, TN 37232, USA; Department of Neurology, Mayo Clinic-Minnesota, Rochester, MN 55905, USA; Department of Neurology, Mayo Clinic-Minnesota, Rochester, MN 55905, USA; Department of Radiology, Mayo Clinic-Minnesota, Rochester, MN 55905, USA; Department of Neurology, Mayo Clinic-Minnesota, Rochester, MN 55905, USA; Department of Health Sciences Research, Mayo Clinic-Minnesota, Rochester, MN 55905, USA; Department of Radiology, Mayo Clinic-Minnesota, Rochester, MN 55905, USA; Department of Neurology, Mayo Clinic-Minnesota, Rochester, MN 55905, USA; Department of Health Sciences Research, Mayo Clinic-Minnesota, Rochester, MN 55905, USA; Department of Neuroscience, Mayo Clinic-Florida, Jacksonville, FL 32224, USA; Department of Clinical Genomics, Mayo Clinic-Florida, Jacksonville, FL 32224, USA; Department of Radiology, Mayo Clinic-Minnesota, Rochester, MN 55905, USA

**Keywords:** Alzheimer’s disease, tau, resistance, resilience, imaging genetics

## Abstract

Tau deposition is a key biological feature of Alzheimer’s disease that is closely related to cognitive impairment. However, it remains poorly understood why certain individuals may be more susceptible to tau deposition while others are more resistant. The recent availability of *in vivo* assessment of tau burden through positron emission tomography provides an opportunity to test the hypothesis that common genetic variants may influence tau deposition. We performed a genome-wide association study of tau-positron emission tomography on a sample of 754 individuals over age 50 (mean age 72.4 years, 54.6% men, 87.6% cognitively unimpaired) from the population-based Mayo Clinic Study of Aging. Linear regression was performed to test nucleotide polymorphism associations with AV-1451 (^18^F-flortaucipir) tau-positron emission tomography burden in an Alzheimer’s-signature composite region of interest, using an additive genetic model and covarying for age, sex and genetic principal components. Genome-wide significant associations with higher tau were identified for rs76752255 (*P *=* *9.91 × 10^−9^, *β* = 0.20) in the tau phosphorylation regulatory gene *PPP2R2B* (protein phosphatase 2 regulatory subunit B) and for rs117402302 (*P  *=* *4.00 × 10^−8^, *β* = 0.19) near *IGF2BP3* (insulin-like growth factor 2 mRNA-binding protein 3). The *PPP2R2B* association remained genome-wide significant after additionally covarying for global amyloid burden and cerebrovascular disease risk, while the *IGF2BP3* association was partially attenuated after accounting for amyloid load. In addition to these discoveries, three single nucleotide polymorphisms within *MAPT* (microtubule-associated protein tau) displayed nominal associations with tau-positron emission tomography burden, and the association of the *APOE* (apolipoprotein E) ɛ4 allele with tau-positron emission tomography was marginally nonsignificant (*P  *=* *0.06, *β* = 0.07). No associations with tau-positron emission tomography burden were identified for other single nucleotide polymorphisms associated with Alzheimer’s disease clinical diagnosis in prior large case–control studies. Our findings nominate *PPP2R2B* and *IGF2BP3* as novel potential influences on tau pathology which warrant further functional characterization. Our data are also supportive of previous literature on the associations of *MAPT* genetic variation with tau, and more broadly supports the inference that tau accumulation may have a genetic architecture distinct from known Alzheimer’s susceptibility genes, which may have implications for improved risk stratification and therapeutic targeting.

## Teaser

Through a genome-wide study of tau positron emission tomography in a population-based sample, Ramanan *et al.* identified novel associations with higher Alzheimer’s-type tau burden for variants in *PPP2R2B* and *IGF2BP3*, both genes which act in pathways with connections to tau phosphorylation.

## Graphical Abstract

**Graphical Abstract:**
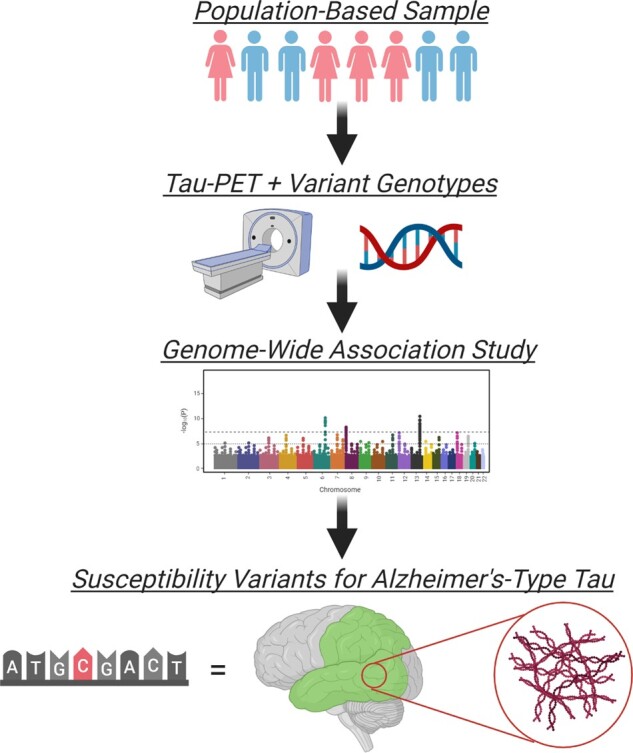

## Introduction

The Alzheimer’s disease biomarker cascade is widely accepted to include neocortical amyloid and tau deposition as prominent features (Jack *et al.*, 2013). Brain amyloidosis is thought to have substantial heritable underpinnings, with numerous studies identifying genetic variants associated with differences in amyloid burden (Thambisetty *et al.*, 2013; Ramanan *et al.*, 2014, 2015; Apostolova *et al.*, 2018; Yan *et al.*, 2018). In comparison however, the genetic influences on tau deposition in Alzheimer’s disease are poorly understood.

The *APOE* (apolipoprotein E) ɛ4 allele, the strongest known genetic risk factor for sporadic Alzheimer’s disease, is well-known to be associated with increased amyloid load (Reiman *et al.*, 2009; Vemuri *et al.*, 2010; Ramanan *et al.*, 2014, 2015) but in population genetics studies appears to display only conditional associations with tau pathology (Farfel *et al.*, 2016; Hohman *et al.*, 2018; Ramanan *et al.*, 2019). A few studies have investigated genetic determinants in relation to levels of cerebrospinal fluid tau (Kauwe *et al.*, 2008; Cruchaga *et al.*, 2013). However, it remains an open question as to why some individuals may be more susceptible to the accumulation of tau pathology in Alzheimer’s disease while others are more resistant, a fundamental unanswered issue given that the burden and topography of tau deposition is closely related to cognitive symptoms in Alzheimer’s disease.

With the recent validation of positron emission tomography ligands for tau (Villemagne *et al.*, 2015; Saint-Aubert *et al.*, 2017), we have an opportunity to noninvasively assess tau pathology *in vivo* in samples large enough for genetic analyses that are conducive for novel discoveries. In this work, we hypothesized that common genetic variants would be associated with susceptibility or resistance to tau deposition in older adults, and conducted a genome-wide association study (GWAS) of tau-positron emission tomography burden to test this hypothesis.

## Materials and methods

### Selection of participants

The Mayo Clinic Study of Aging (MCSA) is a population-based prospective study of older individuals residing in Olmsted County, Minnesota (Roberts *et al.*, 2008; Petersen *et al.*, 2010). Starting in 2004, individuals between 70 and 89 years old were identified for recruitment using the Rochester Epidemiology Project medical records linkage system (Rocca *et al.*, 2012; St Sauver *et al.*, 2012). In 2012, the study was extended to include those aged 50 and older, and starting in 2015, tau-positron emission tomography was added to the MCSA. Clinical data (through questionnaires and in-person history), neuropsychological assessment, neuroimaging and blood laboratory tests were assessed at selected visits. Clinical diagnoses were made by a consensus panel, incorporating all available information. All study protocols were approved by the Mayo Clinic and Olmsted Medical Center Institutional Review Boards and written informed consent was obtained from all participants or their surrogates. Our inclusion criteria included individuals over age 50 with tau-positron emission tomography and genome-wide genotype data.

### Demographic and clinical data

Age, sex, years of education, the number of active medications, and scores on the Mini Mental Status Exam, and Clinical Dementia Rating Sum of Boxes were ascertained for each patient at clinical visit. As a measure of cerebrovascular disease risk, an index score of chronic late-life cardiac, vascular and metabolic conditions was ascertained from health care records as a summation of the presence or absence of hypertension, hyperlipidemia, cardiac arrhythmias, coronary artery disease, congestive heart failure, diabetes and stroke (Vemuri *et al.*, 2017*a*). The Charlson Comorbidity Index was used as a measure of overall health and risk of near-term mortality (Charlson *et al.*, 1987).

### Genetic data

Genomic DNA were extracted from stored peripheral blood samples acquired at the baseline visit for 1783 MCSA participants. Genome-wide genotyping for 658 805 single nucleotide polymorphisms (SNPs) was performed on these DNA samples using the Illumina Infinium Global Screening Array-24 v2.0. This data underwent standard quality control procedures (Anderson *et al.*, 2010) using PLINK version 1.9 (Purcell *et al.*, 2007; Chang *et al.*, 2015). Specifically, SNPs were removed from analysis due to call rate <95% (0 SNPs), monomorphic genotype (100 759 SNPs), minor allele frequency (MAF) <1% (46 843 SNPs), or Hardy–Weinberg equilibrium *P  *<* *1 × 10^−5^ (5067 SNPs). Subjects were removed from analysis due to call rate <98% (0 subjects), sex discordance with clinical data (3 subjects), heterozygosity rate (0 subjects) or batch effects (0 subjects).

For relatedness and population ancestry and structure analyses, SNP pruning was performed including restriction to autosomal SNPs with call rate >95%, MAF >5%, Hardy–Weinberg equilibrium *P  *>* *1 × 10^−5^, exclusion of LCT, HLA and polymorphic inversion regions, and linkage disequilibrium (LD) pruning using a 1000 SNP sliding window to select SNPs with *r*^2^ < 0.2. Twenty-eight subjects were removed due to non-Caucasian ancestry as determined using STRUCTURE version 2.3.4, and using 585 samples from the 1000 Genomes Project as population anchors. Twenty-five pairs of individuals exhibited significant relatedness, defined as PLINK identity by descent PI_HAT >0.25 (Zeng *et al.*, 2015), and therefore one individual from each pair was excluded based on the following criteria, in order of priority: (i) missing tau or amyloid positron emission tomography data; (ii) clinical diagnosis as cognitively unimpaired (as compared to mild cognitive impairment or dementia); (iii) lower call rate. *APOE* ɛ2/ɛ3/ɛ4 allele status determined through genotyping of its defining SNPs (rs429358 and rs7412) on the Illumina chip displayed 100% concordance with allele status previously determined in the MCSA sample via standard restriction digest methods (Hixson and Vernier, 1990). Principal component analysis using SNPRelate version 1.22 (Zheng *et al.*, 2012), identified no significant effects of population stratification within the final dataset. As a conservative measure to account for any potential confounding effects of population structure, the first five principal component eigenvectors were utilized as covariates in all genetic analyses. Following these procedures, data passing quality control was available for 506 136 SNPs and 1727 MCSA participants.

Data from genome-wide imputation were used in this study to evaluate genomic regions and variants of interest which were not directly assayed by the GWAS chip. Prior to imputation, the orientation of all genotyped markers in relation to the plus strand alignment of the Haplotype Reference Consortium reference panel (McCarthy *et al.*, 2016) was verified. Imputation was performed for all autosomal chromosomes with Minimac version 4-1.0.2 via the Michigan Imputation Server (Das *et al.*, 2016) and using default phasing and other recommended quality control parameters and the EUR reference population. Following imputation and removal of monomorphic variants, data was available for 18 873 872 SNPs across the genome. After application of quality control filters as applied to the GWAS chip dataset (SNP call rate < 95%, MAF < 1% and Hardy–Weinberg equilibrium *P  *<* *1 × 10^−5^, and subject call rate < 98%) and removal of SNPs with ambiguous alignment or no identifying rs number, 6 417 232 SNPs were available for analysis. Imputation quality for the dataset was high based on the squared correlation coefficient between imputed allele dosages and masked genotypes (*r*^2^) being 0.992. This imputed data was used for fine-mapping of regions (250 kb upstream and downstream) surrounding SNPs with genome-wide significant association in the GWAS and to analyse a hypothesis-driven list of SNPs previously associated with Alzheimer’s disease clinical diagnosis in large case–control studies through the International Genomics of Alzheimer’s Project (Lambert *et al.*, 2013; Kunkle *et al.*, 2019).

### Neuroimaging data

The acquisition, processing and summary measure details for positron emission tomography imaging performed on MCSA study participants are described in detail elsewhere (Jack *et al.*, 2017). All analyses utilized an in-house fully automated image processing pipeline with 43 atlas-defined regions of interest (ROIs) propagated from an MRI template. Positron emission tomography tracer choice was based on longstanding in-house protocols to maintain consistency.

Tau-positron emission tomography was performed with AV-1451 (^18^F-flortaucipir), synthesized on site using the precursor compound supplied by Avid Radiopharmaceuticals (Xia *et al.*, 2017; Lowe *et al.*, 2019). Regional tau burden was computed from median tracer uptake in each ROI divided by the cerebellar crus grey matter ROI to yield a standardized uptake value ratio (SUVR). The primary outcome measure for this study was tau burden in a composite meta-ROI, computed using median tau uptake in the entorhinal, amygdala, parahippocampal, fusiform and inferior and middle temporal ROIs, divided by the cerebellar crus grey matter ROI (Jack *et al.*, 2017). For comparison, *post hoc* models additionally used as outcomes tau burden in the entorhinal and inferior temporal cortices, regions known to exhibit early and prominent tau pathology in Alzheimer’s disease (Johnson *et al.*, 2016; [Bibr fcaa159-B75][Bibr fcaa159-B77]). Where individuals had multiple longitudinal tau positron emission tomography scans, the first scan was used for the cross-sectional analyses performed in this study. Tau positivity (versus negativity) was defined by meta-ROI SUVR ≥1.25 as previously described (Jack *et al.*, 2019).

Amyloid-positron emission tomography imaging, completed during the same visit as tau-positron emission tomography for all but one individual in the sample, was performed using Pittsburgh compound-B (Klunk *et al.*, 2004). Global cortical amyloid load was computed for each participant from median tracer uptake in the prefrontal, orbitofrontal, parietal, temporal, anterior cingulate and posterior cingulate/precuneus ROIs, divided by the median uptake in the cerebellar crus grey matter ROI. Amyloid positivity (versus negativity) was defined by global cortical SUVR ≥ 1.48 as previously described (Lowe *et al.*, 2019).

### Statistical analyses

Distributions for all neuroimaging biomarkers were assessed using scatter plots and histograms. We elected against the use of a data transformation for the tau- and amyloid-positron emission tomography phenotypes in view of their expected non-normal distribution in a large population-based sample predominated by cognitively unimpaired older adults. Genetic analyses were performed using PLINK version 1.9. Association tests for each SNP with tau-positron emission tomography burden were conducted using linear regression under an additive genetic model and including age at tau-positron emission tomography, sex and the first five genetic principal component eigenvectors as covariates.

#### Hypothesis-driven genetic variant associations with tau-positron emission tomography

In the initial, hypothesis-driven phase of this study, we assessed for associations of variants previously demonstrated as relevant for Alzheimer’s disease, including the *APOE* ɛ2 and ɛ4 alleles and 43 SNPs which displayed genome-wide significant associations with a clinical diagnosis of probable Alzheimer’s disease in the largest available case–control studies (Lambert *et al.*, 2013; Kunkle *et al.*, 2019) through the International Genomics of Alzheimer’s Project consortium (Supplementary Table 1). Imputation was used to determine genotypes for any SNPs not directly assayed by the chip array, with all but one SNP (*HLA-DRB1* rs9271058) successfully imputed after quality control. In this phase we also tested for any associations within chip genotype data for 31 SNPs in *MAPT* (microtubule-associated tau protein) given the direct biological relevance of this gene to tau deposition (Pittman *et al.*, 2006). For these hypothesis-driven analyses, a significance threshold of *P  *<* *6.58 × 10^−4^ (0.05/76 variants) was used.

#### GWAS of tau-positron emission tomography

Next, we performed a GWAS to discover novel SNP associations with tau-positron emission tomography burden. In this study phase, a conservative significance threshold of *P  *<* *5 × 10^−8^ was used based on a Bonferroni correction of one million independent tests (Pe'er *et al.*, 2008). Standardized beta coefficients were generated to describe effect sizes, representing the additive change in standardized tau-positron emission tomography burden (mean = 0, standard deviation = 1) corresponding to each additional minor allele. Manhattan and Q-Q plots were generated using Haploview version 4.2 (Barrett *et al.*, 2005), and regional Manhattan plots were generated using the web-based tool LocusZoom (Pruim *et al.*, 2010). Public databases were utilized for LD calculations based on reference population data, including LDLink (Machiela and Chanock, 2015) and Ensembl version 98 (Hunt *et al.*, 2018).

#### Post-GWAS: complementary models

SPSS Statistics version 22.0 (IBM Corp., Armonk, NY) and SAS version 9.4 (SAS Institute, Inc, Cary, NC) were used for other analyses to extend and complement the GWAS. To assess for potential confounding factors impacting significant SNP associations, demographic and clinical variables were analysed for genotypic group differences using ANCOVA models for continuous variables and logistic regression for categorical variables. Post-GWAS linear and logistic regression models were used to assess the robustness of significant SNP associations, including via the application of additional covariates (*APOE* ɛ4 status, cardiovascular and metabolic condition, global amyloid burden) and stratification of the sample by amyloid or tau status (positive versus negative). Regression models using stepwise forward entry were used to examine the independent variance explained by individual target SNPs after accounting for age, sex, the first five genetic principal components, and global amyloid burden.

#### Post-GWAS: voxel-wise analyses

Voxel-wise analyses were performed using SPM version 12 (Wellcome Trust Centre for Neuroimaging) to characterize the whole-brain spatial distribution of associations for target SNPs, as previously described (Ramanan *et al.*, 2015). For these analyses, age, sex and the first five genetic principal components were included as covariates in an ANCOVA model. A study-specific explicit grey matter mask was used, and results were displayed at a voxel-wise significance threshold of family-wise error-corrected *P  *<* *0.05 with minimum cluster size (k) = 100 voxels. Surface renderings of the voxel-level results were generated for visualization. To ensure that voxel-wise tau associations were not driven by partial volume effects or neurodegeneration, for comparison we tested for associations of these SNPs with tau when correcting for partial volume and separately tested for voxel-level grey matter SNP associations.

#### Post-GWAS: fine-mapping and functional annotation

To fine-map top association signals from the GWAS and screen for proximal multi-SNP combinations tagging those variants, we performed exploratory haplotype proxy analyses in PLINK version 1.07. Using regional imputed data flanking SNPs with genome-wide significant associations, haplotype combinations of 2–5 SNPs in strong LD with the top SNP and with MAF >1% were scanned over maximum windows of 500 SNPs and 250 kb and tested for association with tau-positron emission tomography, covarying for age, sex and the first five genetic principal components. Separately, a variety of public databases were utilized for functional annotation of variants of interest, with the goals of assessing potential SNP-gene relationships as well as LD and tissue-specific gene expression patterns. These resources included LDLink (Machiela and Chanock, 2015), Ensembl version 98 (Hunt *et al.*, 2018), the Human Protein Atlas (Uhlen *et al.*, 2015) and the Single Cell Atlas of the Entorhinal Cortex in Human Alzheimer’s disease database (Grubman *et al.*, 2019).

#### Data availability

Data from this study are available from the authors upon reasonable request.

## Results

### Sample characteristics

The GWAS sample included 754 individuals over 50 years of age with tau-positron emission tomography imaging, with mean age 71.9 years (standard deviation 10.4 years) and with men comprising 55% of the sample (Table 1). The preponderance of individuals in the sample was cognitively unimpaired (87.6%). Comparable to previous estimates of *APOE* allele frequency in individuals of European descent (Heffernan *et al.*, 2016), 29% of the GWAS sample carried at least one ɛ4 allele. A majority of individuals were classified as amyloid negative by positron emission tomography (61%).

**Table 1:** Characteristics of the study sample

Characteristic	**All subjects** ***N* = 754** **Mean (SD) or number (%)**	**Age < 65** ***N* = 197** **Mean (SD) or number (%)**	**65 ≤ Age < 80** ***N* = 360** **Mean (SD) or number (%)**	**Age ≥ 80** ***N* = 197** **Mean (SD) or number (%)**
Age (years)	71.9 (10.4)	59.1 (3.7)	72.4 (4.4)	85.7 (3.9)
Sex	412 (55%) men 344 (45%) women	110 (56%) men 87 (44%) women	187 (52%) men 173 (48%) women	115 (58%) men 82 (42%) women
Education (years)	14.8 (2.6)	15.2 (1.2)	14.8 (2.6)	14.3 (2.9)
CMC	2.0 (1.6)	1.1 (1.2)	2.0 (1.4)	2.9 (1.6)
Mini mental status exam score	28.3 (1.7)	29.1 (1.0)	28.4 (1.6)	27.5 (2.2)
Clinical diagnosis^a^	659 (87.6%) CU 75 (10.0%) MCI 18 (2.4%) DEM	187 (95.4%) CU 9 (4.6%) MCI 0 (0%) DEM	324 (90.0%) CU 31 (8.6%) MCI 5 (1.4%) DEM	148 (75.5%) CU 35 (17.9%) MCI 13 (6.6%) DEM
Meta tau-PET SUVR	1.21 (0.13)	1.16 (0.09)	1.21 (0.11)	1.26 (0.17)
Tau status via PET	549 (72.8%) NEG 205 (27.2%) POS	178 (90.4%) NEG 19 (9.6%) POS	261 (72.5%) NEG 99 (27.5%) POS	110 (55.8%) NEG 87 (44.2%) POS
Amyloid PET SUVR	1.60 (0.44)	1.37 (0.13)	1.57 (0.37)	1.90 (0.56)
Amyloid status via PET^b^	460 (61%) NEG 293 (39%) POS	175 (88.8%) NEG 22 (11.2%) POS	221 (61.6%) NEG 138 (38.4%) POS	64 (32.5%) NEG 133 (67.5%) POS

a Clinical diagnosis unavailable for two individuals in the sample.

b Amyloid PET imaging unavailable for one individual in the sample.

CMC, index score of cardiovascular and metabolic conditions (range: 0–7); CU, cognitively unimpaired; DEM, dementia; MCI, mild cognitive impairment; NEG, negative; POS, positive.

### Hypothesis-driven analyses of Alzheimer’s disease risk loci and *MAPT* with tau-positron emission tomography burden

Neither the *APOE* ɛ2 nor ɛ4 allele demonstrated association with tau burden in the primary meta ROI, with the summary statistics for the ɛ4 allele being marginally nonsignificant prior to correction for multiple comparisons (*P  *=* *0.06, *β*  =  0.07). These results did not appreciably change when using a dominant ( versus additive) genetic model. A significant association of *APOE* ɛ4 with entorhinal cortex tau-positron emission tomography burden (*P  *=* *3.30 × 10^−5^, *β*  =  0.14) and a nominal association with inferior temporal cortex tau-positron emission tomography burden (*P  *=* *0.03, *β*  =  0.08) were identified, both of which were completely attenuated after covarying for global amyloid burden, consistent with prior analyses of a smaller sample from the MCSA cohort (Ramanan *et al.*, 2019). Three SNPs within *MAPT* displayed nominal associations with tau burden, including rs1467967 which tags a common *MAPT* haplotype (Heckman *et al.*, 2019), though none survived correction for multiple comparisons (Supplementary Table 1). No nominal associations with tau were identified for any of the SNPs reported as associated with Alzheimer’s disease clinical diagnosis in prior large consortium case–control studies (Supplementary Table 1).

### GWAS discovers novel SNP associations with tau-positron emission tomography burden

**Figure 1:**
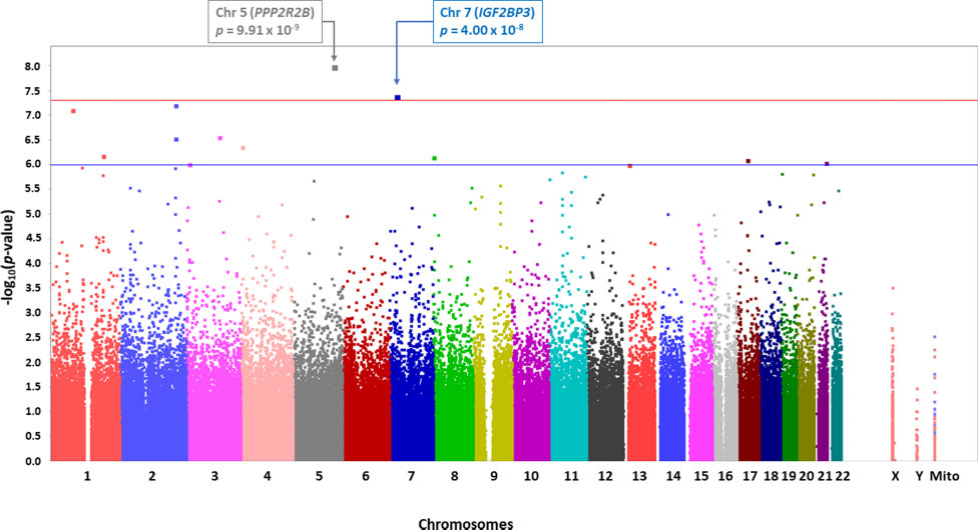
**Manhattan plots for the GWAS of tau-positron emission tomography burden.** Observed −log_10_*P*-values are displayed (*y*-axis) for all single SNPs tested in the GWAS. Genome-wide significant associations (*P* < 5 × 10^−8^; red line) with tau-positron emission tomography burden were identified on chromosomes 5 (rs76752255) in *PPP2R2B* and on chromosome 7 (rs117402302) near *IGF2BP3*. Suggestive associations (*P* < 1 × 10^−6^; blue line) were identified on additional chromosomes.

There was no evidence of spurious systematic inflation of *P*-values in the GWAS due to population stratification or other confounding factors (*λ*  =  1.00; Supplementary Fig. 1). Novel genome-wide significant associations with tau-positron emission tomography burden were identified for rs76752255 (*P  *=* *9.91 × 10^−9^, *β*  =  0.20), an intronic SNP in *PPP2R2B* (protein phosphatase 2, regulatory subunit B, beta) on chromosome 5, and rs117402302 (*P  *=* *4.00 × 10^−8^, *β*  =  0.19), an intergenic SNP near (11 kb) *IGF2BP3* (insulin like growth factor 2 mRNA-binding protein 3) on chromosome 7 (Fig. 1). Strong association signals (*P  *<* *1 × 10^−6^) not meeting the stringent threshold for genome-wide significance were identified for 14 SNPs (Supplementary Table 2). These included rs11722856 (*JAKMIP1*; janus kinase and microtubule interacting protein 1) and rs117603268 (*DLGAP2*; DLG-associated protein 2), both within genes known to have neuronal functions and enriched brain expression. Following regional imputation using a 500 kb window, a nearby *IGF2BP3* intronic SNP (rs138919567) in strong LD with rs117402302 showed similarly strong association (Fig. 1B), while the LD profiles and association statistics of chromosome 5 SNPs flanking *PPP2R2B* rs76752255 were modest (Fig. 1C).

**Figure 2:**
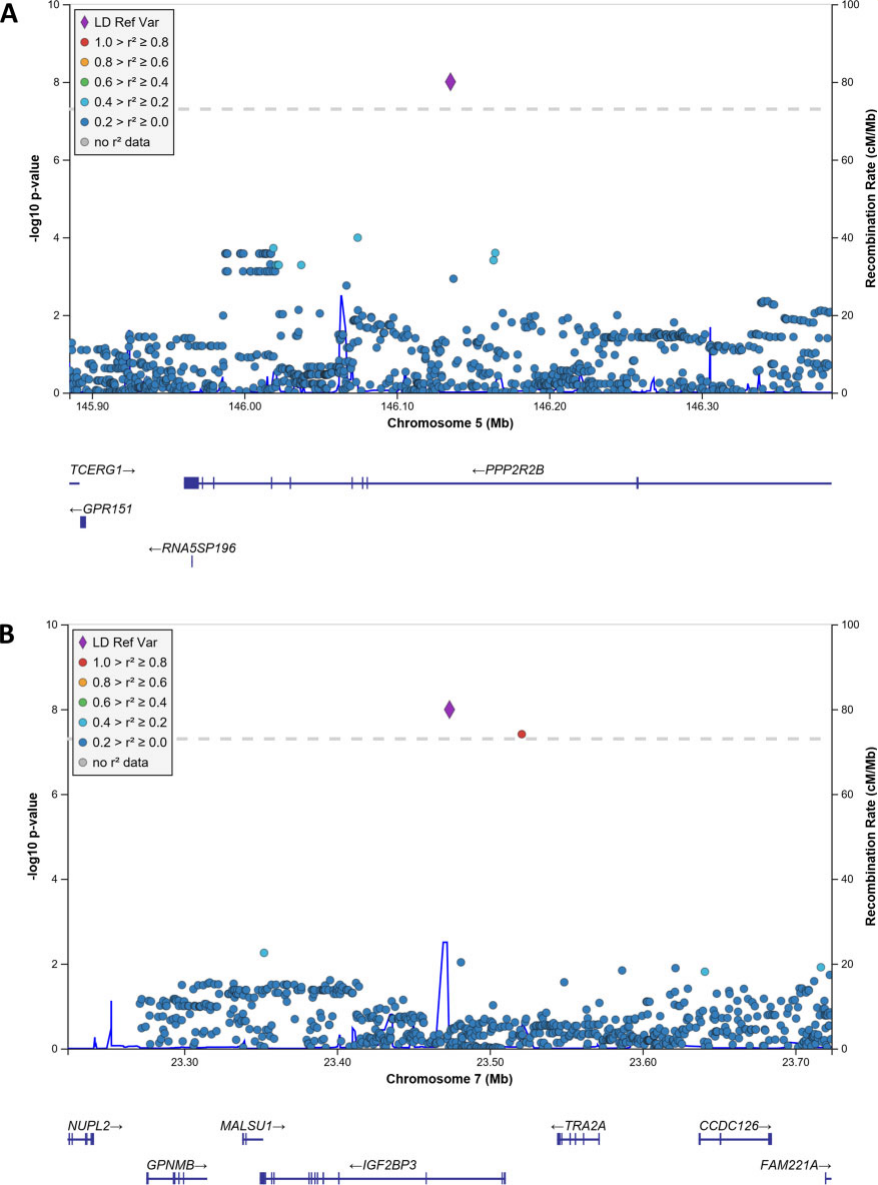
**Regional manhattan plots for the GWAS of tau-positron emission tomography burden.** All genotyped and imputed SNPs within a 500 kb region surrounding *PPP2R2B* rs76752255 (**A**) and *IGF2BP3* rs117402302 (**B**) are plotted based on their association *P*-values, NCBI build 37 genomic position, and recombination rates calculated from the 1000 Genomes Project reference data. The colour scale of *r*^2^ values is used to label SNPs based on their degree of LD with the target SNP. Top SNP associations in the region are labelled. Genes in the region are labelled below the plots, with arrows denoting 5′-to-3′ orientation. Plots were created using the LocusZoom software suite.

#### *PPP2R2B* rs76752255-C and *IGF2BP3* rs117402302 are associated with susceptibility to tau deposition

**Figure 3:**
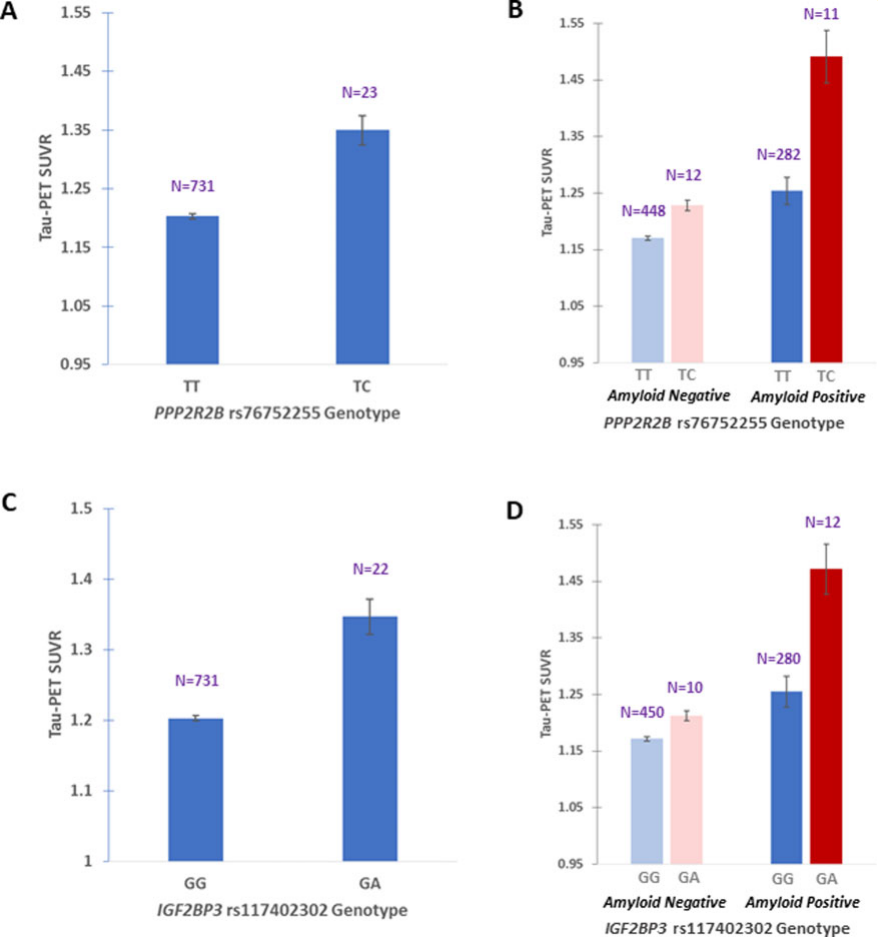
**Associations of *PPP2R2B* rs76752255 and *IGF2BP3* rs117402302 with higher tau.** Tau-positron emission tomography SUVR data for a composite ‘meta’ ROI are shown in relation to *PPP2R2B* rs76752255 and *IGF2BP3* rs117402302 genotype. (**A**) and (**C**) In the full study sample, the rs76752255-TC and rs117402302-GA genotypes were associated with higher tau-positron emission tomography burden. (**B**) and (**D**) In *post hoc* stratified analyses, these alleles demonstrated the same direction of association (protective) for tau-positron emission tomography burden regardless of amyloid status, though the magnitude of SUVR difference was relatively greater in amyloid-positive individuals than in amyloid-negative individuals. One individual from the full study sample was not included in these analyses due to not having an available amyloid-positron emission tomography scan.

The MAFs for *PPP2R2B* rs76752255-C and *IGF2BP3* rs117402302-A (1.5%/1.5%) were comparable to their frequencies in European reference populations (2.0%/2.5%) aggregated in dbSNP (Sherry *et al.*, 2001). Individuals with the *PPP2R2B* rs76752255-TC and *IGF2BP3* rs117402302-GA genotypes each displayed higher levels of tau compared to their rs75546066-TT and *IGF2BP3* rs117402302-GG counterparts (Fig. 3A and C and Supplementary Fig. 2). For comparison with the primary meta-ROI outcome, robust association signals were also observed in the inferior temporal (rs76752255: *P  *=* *8.79 × 10^−9^, *β*  =  0.20; rs117402302: *P  *=* *2.44 × 10^−8^, *β*  =  0.19) and entorhinal (rs76752255: *P  *=* *7.60 × 10^−5^, *β*  =  0.14; rs117402302: *P  *=* *8.55 × 10^−6^; *β*  =  0.15) cortex ROIs. No individuals in the study sample were homozygous for either rs76752255-C or rs117402302-A, and only two individuals had the minor allele for both SNPs. After accounting for age, sex, genetic principal components and global amyloid burden, which together explained 27.9% of the phenotypic variance, *PPP2R2B* rs76752255 and *IGF2BP3* rs117402302 accounted for an additional 3.4% and 1.6% of the variance in tau-positron emission tomography, respectively, based on stepwise entry linear regression.

### Confounders do not explain the associations of *PPP2R2B* rs76752255 and *IGF2BP3* rs117402302 with higher tau-positron emission tomography burden

The associations of *PPP2R2B* rs76752255 and *IGF2BP3* rs117402302 with higher tau remained genome-wide significant when including *APOE* ɛ4 (*P  *=* *7.94 × 10^−9^; *β*  =  0.20, and *P  *=* *2.81 × 10^−8^; *β*  =  0.19, respectively) or cardiovascular and metabolic condition (*P  *=* *4.31 × 10^−9^; *β*  =  0.20, and *P  *=* *4.63 × 10^−8^; *β*  =  0.19, respectively) as additional covariates to the main regression model. When global amyloid burden was included as a covariate, the association of *PPP2R2B* rs76752255 remained genome-wide significant (*P  *=* *2.17 × 10^−9^; *β*  =  0.19), while the association of *IGF2BP3* rs117402302 was partially attenuated (*P  *=* *7.81 × 10^−6^; *β*  =  0.14). *Post hoc* analyses stratified by amyloid-positron emission tomography status (positive versus negative) revealed the same direction of association for these SNPs regardless of amyloid status, though the magnitudes of tau-positron emission tomography SUVR difference were greater in amyloid positive individuals (Fig. 3B and D). Based on logistic regression, carriers of *PPP2R2B* rs76752255-C were more likely than non-carriers to be tau-positive [*P  *=* *0.01, OR = 3.24 (95% CI 1.27–8.23)] when covarying for age, sex, genetic principal components and global amyloid burden. No allelic differences in tau status were identified for IGF2BP3 rs1174024302 after accounting for global amyloid burden.

Overall, carriers of rs76752255-C were significantly more impaired than non-carriers based on the Clinical Dementia Rating scale (*P  *<* *0.001) despite having no or marginally significant differences in age, sex, education, cerebrovascular disease risk and brain amyloid burden (Supplementary Table 3). Carriers of rs117402302-A similarly displayed greater impairment than non-carriers on the Clinical Dementia Rating (*P  *<* *0.001) and had higher amyloid burden (*P  *<* *0.001) but no differences in age, sex, education or cerebrovascular disease risk. When a stronger relatedness threshold was used (PI_HAT >0.125) resulting in removal of an additional subject from analysis, the associations of *PPP2R2B* rs76752255 (*P  *=* *1.01 × 10^−8^; *β*  =  0.20) and *IGF2BP3* rs117402302 (*P  *=* *4.09 × 10^−8^; *β*  =  0.19) with higher tau remained genome-wide significant with unchanged beta weights, indicating no spurious effect from cryptic relatedness in the primary sample.

### Voxel-wise analyses find associations of *PPP2R2B* rs76752255 and *IGF2BP3* rs117402302 with higher tau-positron emission tomography burden

**Figure 4:**
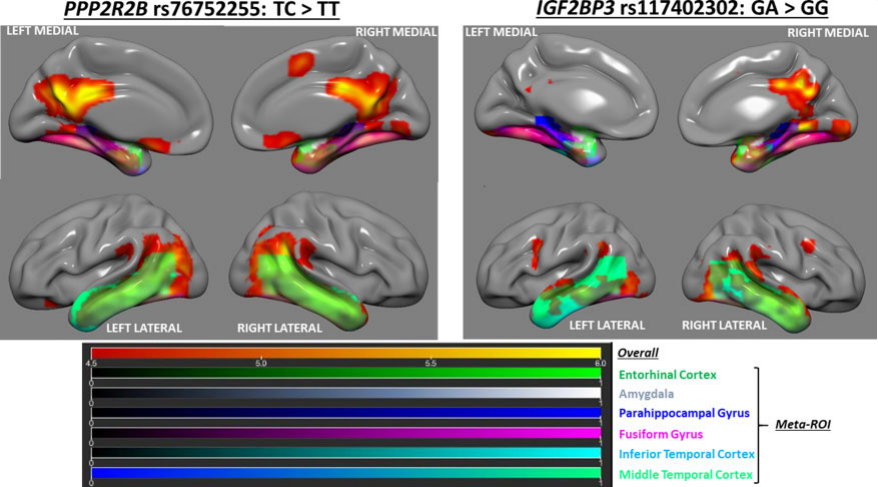
**Voxel-wise whole brain analyses of the associations of *PPP2R2B* rs76752255 and *IGF2BP3* rs117402302 with higher tau.** Glass brain surface renderings from whole-brain voxel-wise analyses are displayed. Intensity scales are shown below, with brighter colours on the primary red-orange-yellow scale indicating progressively stronger association signals, and with individual regions comprising the composite ‘meta-ROI’ overlayed and denoted by distinct colour scales as labelled. Significant clusters of association are shown for *PPP2R2B* rs76752255-C (left panel) and *IGF2BP3* rs117402302 (right panel) with higher tau-positron emission tomography burden, with predominant effects observed in the medial, lateral and inferior temporal and posterior cingulate regions.

We performed whole-brain voxel-wise analyses to assess the broader spatial distribution for the associations of the two target SNPs with tau-positron emission tomography burden. Compared to minor allele noncarriers, individuals with either the *PPP2R2B* rs76752255-C or *IGF2BP3* rs117402302-A alleles displayed higher tau on a voxel level in an Alzheimer’s disease-pattern distribution, with predominant clusters in the medial, lateral and inferior temporal and posterior cingulate regions (Fig. 4 and Supplementary Table 5). The results were unchanged when additionally correcting for partial volume, and corresponding voxel-wise grey matter analyses revealed no significant differences between minor allele carriers versus noncarriers for either SNP, indicating that the tau associations were not driven by partial volume effects related to neurodegeneration. For comparison, voxel-wise analyses for *APOE* ɛ4 showed only modest focal medial temporal signal (Supplementary Fig. 3), in contrast to the robust Alzheimer’s disease pattern of association seen for the *PPP2R2B* and *IGF2BP3* SNPs identified through GWAS.

### Proxy analyses suggest that *PPP2R2B* rs76752255 may tag a nearby haplotype

Using regional imputed data to maximize genomic coverage, we screened for haplotypic multi-SNP combinations that may tag the top association signals from the GWAS. Within the region surrounding *PPP2R2B* rs76752255, despite no single SNPs displaying substantial LD with rs76752255, 6 haplotypes (combinations of 2–5 SNPs) exhibited evidence for serving as proxies (*r*^2^ > 0.8). Among these, the haplotype consisting of rs77205756-T/rs17105275-T displayed strong LD with rs76752255 and association with the tau-positron emission tomography phenotype at an even more robust significance level (*P  *=* *5.20 × 10^−11^, *r*^2^ = 0.83 with rs76752255, *β*  =  0.17, frequency = 1.7%), suggesting that the GWAS discovery in *PPP2R2B* may be tagging a nearby haplotype. In contrast, no haplotypes within the region of *IGF2BP3* rs117402302/rs138919567 were found to be proxies for these SNPs.

## Discussion

To our knowledge, this study of a population-based sample of older adults represents the first reported GWAS of tau-positron emission tomography burden. Using this approach, we discovered novel associations of *PPP2R2B* and *IGF2BP3* variants with higher tau deposition in older adults. These findings advance extant knowledge about the potential heritable factors associated with tau pathology in older adults, and highlight the importance of accounting for differential susceptibility and resistance to tau deposition when considering risk of developing Alzheimer’s disease dementia. Our data also support the inference that the genetic and molecular underpinnings of tau deposition in Alzheimer’s disease may be distinct from established Alzheimer’s disease susceptibility genes, arguing for a broadening of thought regarding models of risk stratification and therapeutic targeting.

The influences on tau deposition in aging and neurodegenerative disease are presumed to be complex and multifactorial. β-amyloidosis is a risk factor for tau accumulation within and outside of the medial temporal lobe (He *et al.*, 2018; Jack *et al.*, 2018, 2019; Pontecorvo *et al.*, 2019; Ramanan *et al.*, 2019). However, a substantial proportion of older individuals who are cognitively unimpaired harbor amyloid pathology (Sperling *et al.*, 2013; Jansen *et al.*, 2015; Petersen *et al.*, 2016), including some individuals who meet biomarker criteria for amyloid positivity but not for tau positivity. These different biomarker profiles result in differences in longitudinal clinical trajectories (Jack *et al.*, 2019). Given that cognitive decline in Alzheimer’s disease is more closely related to tau accumulation than to amyloid accumulation (Nelson *et al.*, 2012; Ossenkoppele *et al.*, 2016; Bejanin *et al.*, 2017), understanding why certain individuals may have relatively more resistance (avoidance) or resilience (coping) against tau deposition may be crucial for improved risk stratification and therapeutic targeting in Alzheimer’s disease (Arenaza-Urquijo and Vemuri, 2018).

Our new findings support the concept that heritable factors can influence susceptibility and resistance to tau pathology. It is noteworthy that individuals with the *PPP2R2B* susceptibility allele in this study displayed significantly higher levels of tau and clinical impairment despite having no or marginally significant differences with their counterparts in age, sex, education, cerebrovascular disease risk, *APOE* ɛ4 status, and brain amyloid burden, supporting the concept that tau-associated genetic variation may impact clinically-relevant Alzheimer’s disease pathophysiology independently from other Alzheimer’s disease dementia-associated risk factors. However, individuals with the *IGF2BP3* susceptibility allele had higher amyloid load compared to their counterparts even though both groups had similar ages, suggesting that this allele may impact multiple components of the Alzheimer’s disease biomarker cascade. The complementary voxel-wise analyses also demonstrated that the whole-brain topography of higher tau burden associated with the *PPP2R2B* and *IGF2BP3* variants was of a frank Alzheimer’s disease pattern, and was not driven by isolated focal signal hotspots or partial volume effects related to neurodegeneration. Collectively, these notions lend additional credence to the theory that Alzheimer’s disease is etiologically complex and that a combination of multiple genetic and lifestyle/environmental factors can influence its key pathophysiologic processes on an individualized basis (Reitz, 2016).

Plausible biological mechanisms can be inferred from existing literature to account for these novel associations. *PPP2R2B* is highly expressed in the brain (Uhlen *et al.*, 2015) and regulates the activity of the PP2A (protein phosphatase 2A) family of enzymes, which serve as a principal factor in controlling the phosphorylation status of tau and whose inhibition or downregulation have been shown to promote tau phosphorylation (Torrent and Ferrer, 2012; Sontag and Sontag, 2014). Triplet expansion in *PPP2R2B* causes spinocerebellar ataxia type 12 (Srivastava *et al.*, 2017), but variation in this gene had not previously been associated with Alzheimer’s disease in humans. Based on an atlas of single cell gene expression from entorhinal cortex tissue samples (Grubman *et al.*, 2019), *PPP2R2B* has lower expression in Alzheimer’s disease compared to controls in numerous cell types, with the strongest association in oligodendrocytes (FDR-corrected *P  *=* *8.92 × 10^−171^). This background supports the potential relevance of genetic variation in *PPP2R2B* for impacting neurodegenerative processes and highlights the value of our GWAS discovery in this region.

*IGF2BP3* has well-known roles in a number of cancers (Lederer *et al.*, 2014), including as a glioblastoma marker (Suvasini *et al.*, 2011). An orthologue of *IGF2BP3* (*IGF2BP1*, also known as IMP1) is known to bind the 3′ untranslated region of tau mRNA (Atlas *et al.*, 2004). More broadly, insulin and insulin-like growth factor pathways represent targets of active interest in Alzheimer’s disease (Arvanitakis *et al*., 2020). This backdrop includes Alzheimer’s disease model system data which support a neuroprotective role for IGFs, and a hypothesis that resistance to this signaling may lead to disinhibition of pro-apoptotic and pro-inflammatory pathways as well as tau phosphorylation related to phosphatidylinositol 3-kinase, protein kinase B (Akt) and glycogen synthase kinase 3 pathways (Benarroch, 2012). Nevertheless, the import of these potential mechanisms in relation to tau in Alzheimer’s disease is still unclear, and our findings suggest that additional study of the *IGF2BP3* locus may be warranted. More broadly, since top association signals may not always be indicative of the true causal genetic change and given that both coding and non-coding variants can exert functional impacts at distance (Kapranov *et al.*, 2007; Consortium, 2012), our findings argue for further study of the wider loci containing rs76752255 and rs117402302 to more precisely characterize the functional architectures of these genomic regions.

Nominal associations with tau burden were identified for 3 SNPs within *MAPT*, including the haplotype-tagging SNP rs1467967 which was previously associated with brain *MAPT* expression (Allen *et al.*, 2014) and levels of cerebrospinal fluid tau (Babić Leko *et al.*, 2018). Mutations in *MAPT* are well-known causes of certain tau-related frontotemporal lobar degenerative and atypical Parkinsonian syndromes (Rademakers *et al.*, 2004; Höglinger *et al.*, 2011; Strang *et al.*, 2019), some of which include tau pathology that can be effectively assessed by AV-1451 tau-positron emission tomography (Spina *et al.*, 2017). Our data provide some additional support for the association of *MAPT* genetic variation with tau burden, and more broadly reinforces prior work suggesting roles for *MAPT* in the pathophysiology of Alzheimer’s disease and other tauopathies (Allen *et al.*, 2014; Desikan *et al.*, 2015; Heckman *et al.*, 2019).

There continues to be strong interest in whether a mechanistic relationship exists between *APOE* and brain parenchymal tau that is independent of amyloid, as has been suggested by analyses of primary tauopathies and selected studies using cellular and animal model systems (Shi *et al.*, 2017; Zhao *et al.*, 2018; Wadhwani *et al.*, 2019). Human *in vivo* imaging studies along the Alzheimer’s disease spectrum have also assessed this issue, which may be impacted by the population being analysed (Ossenkoppele *et al.*, 2016; Ramanan *et al.*, 2019). The findings from this study support the hypothesis that in the general population of older adults, the association of *APOE* with tau accumulation would appear to be amyloid-dependent and at minimum may be substantially weaker than its association with amyloidosis.

In addition, our study found no associations with tau-positron emission tomography burden for other SNPs previously associated with clinically defined probable Alzheimer’s disease risk in large case–control studies. Given that several of these genetic variants have displayed associations with amyloid-positron emission tomography burden in prior studies (Ramanan *et al.*, 2014, 2015; Apostolova *et al.*, 2018), our findings suggest that the genetic background influencing tau pathology may meaningfully differ from the set of genes typically proposed as top loci for Alzheimer’s disease clinical diagnosis. This seeming dichotomy argues for a broadening of thought about potential targets for risk prediction and clinically meaningful interventions in Alzheimer’s disease, with particular mind to the potential value of multi-pronged therapy given that factors both related and unrelated to amyloid are likely to influence the disease.

This work has limitations. Although larger than many of the published genome-wide studies of amyloid-positron emission tomography and other Alzheimer’s disease endophenotypes, our GWAS of tau-positron emission tomography included a relatively modest sample size compared to non-biomarker-based studies. As such, the novel associations reported in this study warrant a degree of caution. In addition, given the novelty of this approach and its setting consisting of a large, single-site, population-based sample with tau-positron emission tomography, no comparable replication cohort was available. As the first study on a new imaging genetics dataset, we focused on directly genotyped SNPs in the GWAS, which is an advantage relative to the robustness of our top findings. However, follow-up studies utilizing genome-wide imputed SNP data will provide higher density genomic coverage and facilitate complementary analytical approaches. The relatively low MAFs and lack of minor allele homozygotes for the top SNPs in our study also limited evaluation for potential interactions with other factors and precluded assessment for dosage effects of these alleles. In addition, the cross-sectional design of our study was not equipped to test whether longitudinal imaging and clinical trajectories differ based on genotype profiles. Although outside the scope of this study, which focused on common genetic variation, genome sequencing data would allow for association testing among rare variants, such as the *APOE* Christchurch variant recently proposed as protective even in the setting of the *PSEN1* (presenilin 1) E280A mutation which causes autosomal dominant Alzheimer’s disease (Arboleda-Velasquez *et al.*, 2019). Furthermore, the AV-1451 tracer measures Alzheimer’s disease-type mixed 3R/4R tau deposits and not isolated 3R or 4R tau aggregates, and the specificity and sensitivity of AV-1451 to Alzheimer’s disease-related tauopathy are not perfect. These features could have import for interpretation of imaging genetics associations using tau-positron emission tomography (Leuzy *et al.*, 2019).

Nevertheless, this study highlights the value of pairing advanced molecular imaging with genetics to illuminate potential mechanisms underlying Alzheimer’s disease pathophysiologic features. In particular, the novel associations of *PPP2R2B* and *IGF2BP3* with susceptibility to tau pathology warrant further investigation through validation studies and functional characterization, and may have significant implications for future risk counseling and therapeutic development.

## Supplementary Material

fcaa159_Supplementary_Data

## Abbreviations

GWAS =: genome-wide association study
IGAP =: International Genomics of Alzheimer’s Project
MAF =: minor allele frequency
MCSA =: Mayo Clinic Study of Aging
ROI =: region of interest
SNP =: single nucleotide polymorphism
SUVR =: standardized uptake value ratio

## References

[fcaa159-B1] Allen M Kachadoorian M Quicksall Z Zou F Chai HS Younkin C , et al Association of MAPT haplotypes with Alzheimer's disease risk and MAPT brain gene expression levels. Alzheimers Res Ther 2014; 6: 39. 25324900 10.1186/alzrt268 PMC4198935

[fcaa159-B2] Anderson CA Pettersson FH Clarke GM Cardon LR Morris AP Zondervan KT. Data quality control in genetic case–control association studies. Nat Protoc 2010; 5: 1564–73. 21085122 10.1038/nprot.2010.116 PMC3025522

[fcaa159-B3] Apostolova LG Risacher SL Duran T Stage EC Goukasian N West JD , et al Associations of the top 20 Alzheimer disease risk variants with brain amyloidosis. JAMA Neurol 2018; 75: 328–41. 29340569 10.1001/jamaneurol.2017.4198 PMC5885860

[fcaa159-B4] Arboleda-Velasquez JF Lopera F O’Hare M Delgado-Tirado S Marino C Chmielewska N , et al Resistance to autosomal dominant Alzheimer's disease in an APOE3 Christchurch homozygote: a case report. Nat Med 2019; 25: 1680–3. 31686034 10.1038/s41591-019-0611-3 PMC6898984

[fcaa159-B5] Arenaza-Urquijo EM Vemuri P. Resistance vs resilience to Alzheimer disease: clarifying terminology for preclinical studies. Neurology 2018; 90: 695–703. 29592885 10.1212/WNL.0000000000005303 PMC5894932

[fcaa159-B6] Arvanitakis Z Wang HY Capuano AW Khan A Taib B Anokye-Danso F , et al Brain insulin signaling, Alzheimer's disease pathology, and cognitive function. Ann Neurol 2020; 88: 513–25. 32557841 10.1002/ana.25826 PMC7722192

[fcaa159-B7] Atlas R Behar L Elliott E Ginzburg I. The insulin-like growth factor mRNA binding-protein IMP-1 and the Ras-regulatory protein G3BP associate with tau mRNA and HuD protein in differentiated P19 neuronal cells. J Neurochem 2004; 89: 613–26. 15086518 10.1111/j.1471-4159.2004.02371.x

[fcaa159-B8] Babić Leko M Willumsen N Nikolac Perković M Klepac N Borovečki F Hof PR , et al Association of MAPT haplotype-tagging polymorphisms with cerebrospinal fluid biomarkers of Alzheimer's disease: a preliminary study in a Croatian cohort. Brain Behav 2018; 8: e01128. 30329219 10.1002/brb3.1128 PMC6236251

[fcaa159-B9] Barrett JC Fry B Maller J Daly MJ. Haploview: analysis and visualization of LD and haplotype maps. Bioinformatics 2005; 21: 263–5. 15297300 10.1093/bioinformatics/bth457

[fcaa159-B10] Bejanin A Schonhaut DR La Joie R Kramer JH Baker SL Sosa N , et al Tau pathology and neurodegeneration contribute to cognitive impairment in Alzheimer's disease. Brain 2017; 140: 3286–300. 29053874 10.1093/brain/awx243 PMC5841139

[fcaa159-B11] Benarroch EE. Insulin-like growth factors in the brain and their potential clinical implications. Neurology 2012; 79: 2148–53. 23170013 10.1212/WNL.0b013e3182752eef

[fcaa159-B12] Chang CC Chow CC Tellier LC Vattikuti S Purcell SM Lee JJ. Second-generation PLINK: rising to the challenge of larger and richer datasets. GigaSci 2015; 4: 7. 10.1186/s13742-015-0047-8 PMC4342193 25722852

[fcaa159-B13] Charlson ME Pompei P Ales KL MacKenzie CR. A new method of classifying prognostic comorbidity in longitudinal studies: development and validation. J Chronic Dis 1987; 40: 373–83. 3558716 10.1016/0021-9681(87)90171-8

[fcaa159-B14] Consortium EP. An integrated encyclopedia of DNA elements in the human genome. Nature 2012; 489: 57–74. 22955616 10.1038/nature11247 PMC3439153

[fcaa159-B15] Cruchaga C Kauwe JS Harari O Jin SC Cai Y Karch CM , et al GWAS of cerebrospinal fluid tau levels identifies risk variants for Alzheimer's disease. Neuron 2013; 78: 256–68. 23562540 10.1016/j.neuron.2013.02.026 PMC3664945

[fcaa159-B16] Das S Forer L Schonherr S Sidore C Locke AE Kwong A , et al Next-generation genotype imputation service and methods. Nat Genet 2016; 48: 1284–7. 27571263 10.1038/ng.3656 PMC5157836

[fcaa159-B17] Desikan RS Schork AJ Wang Y Witoelar A Sharma M McEvoy LK , et al Genetic overlap between Alzheimer's disease and Parkinson's disease at the MAPT locus. Mol Psychiatry 2015; 20: 1588–95. 25687773 10.1038/mp.2015.6 PMC4539304

[fcaa159-B18] Farfel JM Yu L De Jager PL Schneider JA Bennett DA. Association of APOE with tau-tangle pathology with and without beta-amyloid. Neurobiol Aging 2016; 37: 19–25. 26481403 10.1016/j.neurobiolaging.2015.09.011 PMC4716785

[fcaa159-B19] Grubman A Chew G Ouyang JF Sun G Choo XY McLean C , et al A single-cell atlas of entorhinal cortex from individuals with Alzheimer’s disease reveals cell-type-specific gene expression regulation. Nat Neurosci 2019; 22: 2087–97. 31768052 10.1038/s41593-019-0539-4

[fcaa159-B20] He Z Guo JL McBride JD Narasimhan S Kim H Changolkar L , et al Amyloid-beta plaques enhance Alzheimer's brain tau-seeded pathologies by facilitating neuritic plaque tau aggregation. Nat Med 2018; 24: 29–38. 29200205 10.1038/nm.4443 PMC5760353

[fcaa159-B21] Heckman MG Brennan RR Labbe C Soto AI Koga S DeTure MA , et al Association of MAPT subhaplotypes with risk of progressive supranuclear palsy and severity of tau pathology. JAMA Neurol 2019; 76: 710–7. 30882841 10.1001/jamaneurol.2019.0250 PMC6563568

[fcaa159-B22] Heckman MG Kasanuki K Brennan RR Labbé C Vargas ER Soto AI , et al Association of MAPT H1 subhaplotypes with neuropathology of Lewy body disease. Mov Disord 2019; 34: 1325–32. 31234228 10.1002/mds.27773 PMC7996001

[fcaa159-B23] Heffernan AL Chidgey C Peng P Masters CL Roberts BR. The neurobiology and age-related prevalence of the epsilon4 allele of apolipoprotein E in Alzheimer's disease cohorts. J Mol Neurosci 2016; 60: 316–24. 27498201 10.1007/s12031-016-0804-x PMC5531868

[fcaa159-B24] Hixson JE Vernier DT. Restriction isotyping of human apolipoprotein E by gene amplification and cleavage with HhaI. J Lipid Res 1990; 31: 545–8. 2341813

[fcaa159-B25] Höglinger GU Melhem NM Dickson DW Sleiman PMA Wang L-S Klei L , et al Identification of common variants influencing risk of the tauopathy progressive supranuclear palsy. Nat Genet 2011; 43: 699–705. 21685912 10.1038/ng.859 PMC3125476

[fcaa159-B26] Hohman TJ Dumitrescu L Barnes LL Thambisetty M Beecham G Kunkle B , et al Sex-specific association of apolipoprotein E with cerebrospinal fluid levels of tau. JAMA Neurol 2018; 75: 989–98. 29801024 10.1001/jamaneurol.2018.0821 PMC6142927

[fcaa159-B27] Hunt SE McLaren W Gil L Thormann A Schuilenburg H Sheppard D , et al Ensembl variation resources. Database (Oxford) 2018; 2018: bay119. 10.1093/database/bay119 PMC6310513 30576484

[fcaa159-B28] Jack CR Jr Knopman DS Jagust WJ Petersen RC Weiner MW Aisen PS , et al Tracking pathophysiological processes in Alzheimer's disease: an updated hypothetical model of dynamic biomarkers. Lancet Neurol 2013; 12: 207–16. 23332364 10.1016/S1474-4422(12)70291-0 PMC3622225

[fcaa159-B29] Jack CR Wiste HJ Botha H Weigand SD Therneau TM Knopman DS , et al The bivariate distribution of amyloid-beta and tau: relationship with established neurocognitive clinical syndromes. Brain 2019; 142: 3230–42. 31501889 10.1093/brain/awz268 PMC6763736

[fcaa159-B30] Jack CR Jr Wiste HJ Schwarz CG Lowe VJ Senjem ML Vemuri P , et al Longitudinal tau PET in ageing and Alzheimer's disease. Brain 2018; 141: 1517–28. 29538647 10.1093/brain/awy059 PMC5917767

[fcaa159-B31] Jack CR Jr Wiste HJ Therneau TM Weigand SD Knopman DS Mielke MM , et al Associations of amyloid, tau, and neurodegeneration biomarker profiles with rates of memory decline among individuals without dementia. JAMA 2019; 321: 2316–25. 31211344 10.1001/jama.2019.7437 PMC6582267

[fcaa159-B32] Jack CR Jr Wiste HJ Weigand SD Therneau TM Lowe VJ Knopman DS , et al Defining imaging biomarker cut points for brain aging and Alzheimer's disease. Alzheimers Dement 2017; 13: 205–16. 27697430 10.1016/j.jalz.2016.08.005 PMC5344738

[fcaa159-B33] Jansen WJ Ossenkoppele R Knol DL Tijms BM Scheltens P Verhey FR , et al Prevalence of cerebral amyloid pathology in persons without dementia: a meta-analysis. JAMA 2015; 313: 1924–38. 25988462 10.1001/jama.2015.4668 PMC4486209

[fcaa159-B34] Johnson KA Schultz A Betensky RA Becker JA Sepulcre J Rentz D , et al Tau positron emission tomographic imaging in aging and early Alzheimer disease. Ann Neurol 2016; 79: 110–9. 26505746 10.1002/ana.24546 PMC4738026

[fcaa159-B35] Kapranov P Willingham AT Gingeras TR. Genome-wide transcription and the implications for genomic organization. Nat Rev Genet 2007; 8: 413–23. 17486121 10.1038/nrg2083

[fcaa159-B36] Kauwe JS Cruchaga C Mayo K Fenoglio C Bertelsen S Nowotny P , et al Variation in MAPT is associated with cerebrospinal fluid tau levels in the presence of amyloid-beta deposition. Proc Natl Acad Sci U S A 2008; 105: 8050–4. 18541914 10.1073/pnas.0801227105 PMC2430357

[fcaa159-B37] Klunk WE Engler H Nordberg A Wang Y Blomqvist G Holt DP , et al Imaging brain amyloid in Alzheimer's disease with Pittsburgh compound-B. Ann Neurol 2004; 55: 306–19. 14991808 10.1002/ana.20009

[fcaa159-B38] Kunkle BW Grenier-Boley B Sims R Bis JC Damotte V Naj AC , et al Genetic meta-analysis of diagnosed Alzheimer's disease identifies new risk loci and implicates Abeta, tau, immunity and lipid processing. Nat Genet 2019; 51: 414–30. 30820047 10.1038/s41588-019-0358-2 PMC6463297

[fcaa159-B39] Lambert JC Ibrahim-Verbaas CA Harold D Naj AC Sims R Bellenguez C , et al Meta-analysis of 74,046 individuals identifies 11 new susceptibility loci for Alzheimer's disease. Nat Genet 2013; 45: 1452–8. 24162737 10.1038/ng.2802 PMC3896259

[fcaa159-B40] Lederer M Bley N Schleifer C Huttelmaier S. The role of the oncofetal IGF2 mRNA-binding protein 3 (IGF2BP3) in cancer. Semin Cancer Biol 2014; 29: 3–12. 25068994 10.1016/j.semcancer.2014.07.006

[fcaa159-B41] Leuzy A Chiotis K Lemoine L Gillberg PG Almkvist O Rodriguez-Vieitez E , et al Tau PET imaging in neurodegenerative tauopathies-still a challenge. Mol Psychiatry 2019; 24: 1112–34. 30635637 10.1038/s41380-018-0342-8 PMC6756230

[fcaa159-B42] Lowe VJ Bruinsma TJ Wiste HJ Min HK Weigand SD Fang P , et al Cross-sectional associations of tau-PET signal with cognition in cognitively unimpaired adults. Neurology 2019; 93: e29–39. 31147421 10.1212/WNL.0000000000007728 PMC6659005

[fcaa159-B43] Machiela MJ Chanock SJ. LDlink: a web-based application for exploring population-specific haplotype structure and linking correlated alleles of possible functional variants. Bioinformatics 2015; 31: 3555–7. 26139635 10.1093/bioinformatics/btv402 PMC4626747

[fcaa159-B44] McCarthy S Das S Kretzschmar W Delaneau O Wood AR Teumer A , et al A reference panel of 64,976 haplotypes for genotype imputation. Nat Genet 2016; 48: 1279–83. 27548312 10.1038/ng.3643 PMC5388176

[fcaa159-B45] Nelson PT Alafuzoff I Bigio EH Bouras C Braak H Cairns NJ , et al Correlation of Alzheimer disease neuropathologic changes with cognitive status: a review of the literature. J Neuropathol Exp Neurol 2012; 71: 362–81. 22487856 10.1097/NEN.0b013e31825018f7 PMC3560290

[fcaa159-B46] Ossenkoppele R Schonhaut DR Scholl M Lockhart SN Ayakta N Baker SL , et al Tau PET patterns mirror clinical and neuroanatomical variability in Alzheimer's disease. Brain 2016; 139: 1551–67. 26962052 10.1093/brain/aww027 PMC5006248

[fcaa159-B47] Pe'er I Yelensky R Altshuler D Daly MJ. Estimation of the multiple testing burden for genomewide association studies of nearly all common variants. Genet Epidemiol 2008; 32: 381–5. 18348202 10.1002/gepi.20303

[fcaa159-B48] Petersen RC Roberts RO Knopman DS Geda YE Cha RH Pankratz VS , et al Prevalence of mild cognitive impairment is higher in men. The Mayo Clinic Study of Aging. Neurology 2010; 75: 889–97. 20820000 10.1212/WNL.0b013e3181f11d85 PMC2938972

[fcaa159-B49] Petersen RC Wiste HJ Weigand SD Rocca WA Roberts RO Mielke MM , et al Association of elevated amyloid levels with cognition and biomarkers in cognitively normal people from the community. JAMA Neurol 2016; 73: 85–92. 26595683 10.1001/jamaneurol.2015.3098 PMC4710552

[fcaa159-B50] Pittman AM Fung HC de Silva R. Untangling the tau gene association with neurodegenerative disorders. Hum Mol Genet 2006; 15: R188–95. 16987883 10.1093/hmg/ddl190

[fcaa159-B51] Pontecorvo MJ Devous MD Kennedy I Navitsky M Lu M Galante N , et al A multicentre longitudinal study of flortaucipir (18F) in normal ageing, mild cognitive impairment and Alzheimer's disease dementia. Brain 2019; 142: 1723–35. 31009046 10.1093/brain/awz090 PMC6536847

[fcaa159-B52] Pruim RJ Welch RP Sanna S Teslovich TM Chines PS Gliedt TP , et al LocusZoom: regional visualization of genome-wide association scan results. Bioinformatics 2010; 26: 2336–7. 20634204 10.1093/bioinformatics/btq419 PMC2935401

[fcaa159-B53] Purcell S Neale B Todd-Brown K Thomas L Ferreira MA Bender D , et al PLINK: a tool set for whole-genome association and population-based linkage analyses. Am J Hum Genet 2007; 81: 559–75. 17701901 10.1086/519795 PMC1950838

[fcaa159-B54] Rademakers R Cruts M van Broeckhoven C. The role of tau (MAPT) in frontotemporal dementia and related tauopathies. Hum Mutat 2004; 24: 277–95. 15365985 10.1002/humu.20086

[fcaa159-B55] Ramanan VK Castillo AM Knopman DS Graff-Radford J Lowe VJ Petersen RC , et al Association of apolipoprotein E varepsilon4, educational level, and sex with tau deposition and tau-mediated metabolic dysfunction in older adults. JAMA Netw Open 2019; 2: e1913909. 31642932 10.1001/jamanetworkopen.2019.13909 PMC6820045

[fcaa159-B56] Ramanan VK Risacher SL Nho K Kim S Shen L McDonald BC , et al GWAS of longitudinal amyloid accumulation on 18F-florbetapir PET in Alzheimer's disease implicates microglial activation gene IL1RAP. Brain 2015; 138: 3076–88. 26268530 10.1093/brain/awv231 PMC4671479

[fcaa159-B57] Ramanan VK Risacher SL Nho K Kim S Swaminathan S Shen L , et al APOE and BCHE as modulators of cerebral amyloid deposition: a florbetapir PET genome-wide association study. Mol Psychiatry 2014; 19: 351–7. 23419831 10.1038/mp.2013.19 PMC3661739

[fcaa159-B58] Reiman EM Chen K Liu X Bandy D Yu M Lee W , et al Fibrillar amyloid-beta burden in cognitively normal people at 3 levels of genetic risk for Alzheimer's disease. Proc Natl Acad Sci U S A 2009; 106: 6820–5. 19346482 10.1073/pnas.0900345106 PMC2665196

[fcaa159-B59] Reitz C. Toward precision medicine in Alzheimer's disease. Ann Transl Med 2016; 4: 107. 27127760 10.21037/atm.2016.03.05 PMC4828743

[fcaa159-B60] Roberts RO Geda YE Knopman DS Cha RH Pankratz VS Boeve BF , et al The Mayo Clinic Study of Aging: design and sampling, participation, baseline measures and sample characteristics. Neuroepidemiology 2008; 30: 58–69. 18259084 10.1159/000115751 PMC2821441

[fcaa159-B61] Rocca WA Yawn BP St. Sauver JL Grossardt BR Melton LJ. History of the Rochester Epidemiology Project: half a century of medical records linkage in a US population. Mayo Clin Proc 2012; 87: 1202–13. 23199802 10.1016/j.mayocp.2012.08.012 PMC3541925

[fcaa159-B62] Saint-Aubert L Lemoine L Chiotis K Leuzy A Rodriguez-Vieitez E Nordberg A. Tau PET imaging: present and future directions. Mol Neurodegen 2017; 12: 19. 10.1186/s13024-017-0162-3 PMC5319037 28219440

[fcaa159-B63] Sherry ST Ward MH Kholodov M Baker J Phan L Smigielski EM , et al dbSNP: the NCBI database of genetic variation. Nucleic Acids Res 2001; 29: 308–11. 11125122 10.1093/nar/29.1.308 PMC29783

[fcaa159-B64] Shi Y Yamada K Liddelow SA Smith ST Zhao L Luo W , et al ApoE4 markedly exacerbates tau-mediated neurodegeneration in a mouse model of tauopathy. Nature 2017; 549: 523–7. 28959956 10.1038/nature24016 PMC5641217

[fcaa159-B65] Sontag JM Sontag E. Protein phosphatase 2A dysfunction in Alzheimer's disease. Front Mol Neurosci 2014; 7: 16. 24653673 10.3389/fnmol.2014.00016 PMC3949405

[fcaa159-B66] Sperling RA Johnson KA Doraiswamy PM Reiman EM Fleisher AS Sabbagh MN , et al Amyloid deposition detected with florbetapir F 18 ((18)F-AV-45) is related to lower episodic memory performance in clinically normal older individuals. Neurobiol Aging 2013; 34: 822–31. 22878163 10.1016/j.neurobiolaging.2012.06.014 PMC3518678

[fcaa159-B67] Spina S Schonhaut DR Boeve BF Seeley WW Ossenkoppele R O'Neil JP , et al Frontotemporal dementia with the V337M MAPT mutation: tau-PET and pathology correlations. Neurology 2017; 88: 758–66. 28130473 10.1212/WNL.0000000000003636 PMC5344079

[fcaa159-B68] Srivastava AK Takkar A Garg A Faruq M. Clinical behaviour of spinocerebellar ataxia type 12 and intermediate length abnormal CAG repeats in PPP2R2B. Brain 2017; 140: 27–36. 27864267 10.1093/brain/aww269

[fcaa159-B69] Strang KH Golde TE Giasson BI. MAPT mutations, tauopathy, and mechanisms of neurodegeneration. Lab Invest 2019; 99: 912–28. 30742061 10.1038/s41374-019-0197-x PMC7289372

[fcaa159-B70] St Sauver JL Grossardt BR Yawn BP Melton LJ Pankratz JJ Brue SM , et al Data resource profile: the Rochester Epidemiology Project (REP) medical records-linkage system. Int J Epidemiol 2012; 41: 1614–24. 23159830 10.1093/ije/dys195 PMC3535751

[fcaa159-B71] Suvasini R Shruti B Thota B Shinde SV Friedmann-Morvinski D Nawaz Z , et al Insulin growth factor-2 binding protein 3 (IGF2BP3) is a glioblastoma-specific marker that activates phosphatidylinositol 3-kinase/mitogen-activated protein kinase (PI3K/MAPK) pathways by modulating IGF-2. J Biol Chem 2011; 286: 25882–90. 21613208 10.1074/jbc.M110.178012 PMC3138258

[fcaa159-B72] Thambisetty M An Y Nalls M Sojkova J Swaminathan S Zhou Y , et al Effect of complement CR1 on brain amyloid burden during aging and its modification by APOE genotype. Biol Psychiatry 2013; 73: 422–8. 23022416 10.1016/j.biopsych.2012.08.015 PMC3535537

[fcaa159-B73] Torrent L Ferrer I. PP2A and Alzheimer disease. CAR 2012; 9: 248–56. 10.2174/156720512799361682 22299660

[fcaa159-B74] Uhlen M Fagerberg L Hallstrom BM Lindskog C Oksvold P Mardinoglu A , et al Proteomics. Tissue-based map of the human proteome. Science 2015; 347: 1260419. 25613900 10.1126/science.1260419

[fcaa159-B75] Vemuri P Lesnick TG Przybelski SA Knopman DS Lowe VJ Graff-Radford J , et al Age, vascular health, and Alzheimer disease biomarkers in an elderly sample. Ann Neurol 2017a; 82: 706–18. 29023983 10.1002/ana.25071 PMC5696029

[fcaa159-B76] Vemuri P Lowe VJ Knopman DS Senjem ML Kemp BJ Schwarz CG , et al Tau-PET uptake: regional variation in average SUVR and impact of amyloid deposition. Alzheimer's Dement 2017b; 6: 21–30. 10.1016/j.dadm.2016.12.010 PMC5257031 28138510

[fcaa159-B77] Vemuri P Wiste HJ Weigand SD Knopman DS Shaw LM Trojanowski JQ , et al Effect of apolipoprotein E on biomarkers of amyloid load and neuronal pathology in Alzheimer disease. Ann Neurol 2010; 67: 308–16. 20373342 10.1002/ana.21953 PMC2886799

[fcaa159-B78] Villemagne VL Fodero-Tavoletti MT Masters CL Rowe CC. Tau imaging: early progress and future directions. Lancet Neurol 2015; 14: 114–24. 25496902 10.1016/S1474-4422(14)70252-2

[fcaa159-B79] Wadhwani AR Affaneh A Van Gulden S Kessler JA. Neuronal apolipoprotein E4 increases cell death and phosphorylated tau release in Alzheimer disease. Ann Neurol 2019; 85: 726–39. 30840313 10.1002/ana.25455 PMC8123085

[fcaa159-B80] Xia C Makaretz SJ Caso C McGinnis S Gomperts SN Sepulcre J , et al Association of *in vivo* [18F]AV-1451 tau PET imaging results with cortical atrophy and symptoms in typical and atypical Alzheimer disease. JAMA Neurol 2017; 74: 427–36. 28241163 10.1001/jamaneurol.2016.5755 PMC5470368

[fcaa159-B81] Yan Q Nho K Del-Aguila JL Wang X Risacher SL Fan KH , et al Genome-wide association study of brain amyloid deposition as measured by Pittsburgh compound-B (PiB)-PET imaging. Mol Psychiatry 2018; doi: 10.1038/s41380-018-0246-7. 10.1038/s41380-018-0246-7 PMC6219464 30361487

[fcaa159-B82] Zeng P Zhao Y Qian C Zhang L Zhang R Gou J , et al Statistical analysis for genome-wide association study. J Biomed Res 2015; 29: 285–97. 26243515 10.7555/JBR.29.20140007 PMC4547377

[fcaa159-B83] Zhao N Liu CC Van Ingelgom AJ Linares C Kurti A Knight JA , et al APOE epsilon2 is associated with increased tau pathology in primary tauopathy. Nat Commun 2018; 9: 4388. 30348994 10.1038/s41467-018-06783-0 PMC6197187

[fcaa159-B84] Zheng X Levine D Shen J Gogarten SM Laurie C Weir BS. A high-performance computing toolset for relatedness and principal component analysis of SNP data. Bioinformatics 2012; 28: 3326–8. 23060615 10.1093/bioinformatics/bts606 PMC3519454

