# Supplementary material for: Variants in PPP2R2B and IGF2BP3 are associated with higher tau deposition
Source: Brain Commun. 2020 Sep 26;2(2):fcaa159. doi: 10.1093/braincomms/fcaa159 (PMC7780444; doi:10.1093/braincomms/fcaa159)
Supplement: fcaa159_Supplementary_Data [file fcaa159_supplementary_data.docx]

ONLINE SUPPLEMENTARY MATERIAL

Variants in *PPP2R2B* and *IGF2BP3* are Associated with Higher Tau Deposition

Vijay K Ramanan, MD, PhD^a^, Xuewei Wang, PhD^b^, Scott A. Przybelski, BA^b^, Sheelakumari Raghavan, PhD^c^, Michael G. Heckman, MS^d^, Anthony Batzler, BS^b^, Matthew L. Kosel, BS^b^, Timothy J. Hohman, PhD^e^, David S. Knopman, MD^a^, Jonathan Graff-Radford, MD^a^, Val J. Lowe, MD^c^, Michelle M. Mielke, PhD^a,b^, Clifford R. Jack Jr., MD^c^, Ronald C. Petersen, MD, PhD^a,b^, Owen A. Ross, PhD^f,g^, and Prashanthi Vemuri, PhD^c^

*^a^Department of Neurology, Mayo Clinic-Minnesota, Rochester, Minnesota, 55905, USA*

*^b^Department of Health Sciences Research, Mayo Clinic-Minnesota, Rochester, Minnesota, 55905, USA*

*^c^Department of Radiology, Mayo Clinic-Minnesota, Rochester, Minnesota, 55905, USA*

*^d^Division of Biomedical Statistics and Informatics, Mayo Clinic-Florida, Jacksonville, Florida, 32224, USA*

*^e^Department of Neurology, Vanderbilt University Medical Center, Nashville, TN, 37232, USA*

*^f^Department of Neuroscience, Mayo Clinic-Florida, Jacksonville, Florida, 32224, USA*

*^g^Department of Clinical Genomics, Mayo Clinic-Florida, Jacksonville, Florida, 32224, USA*

Corresponding Authors: Vijay K Ramanan, MD, PhD

Department of Neurology, Mayo Clinic

200 First Street SW, Rochester, MN 55905

Phone: (507)293-1283

Email: Ramanan.Vijay@mayo.edu

Prashanthi Vemuri, PhD

Department of Radiology, Mayo Clinic

200 First Street SW, Rochester, MN 55905

Phone: (507)538-0761

Email: [Vemuri.Prashanthi@mayo.edu](mailto:Vemuri.Prashanthi@mayo.edu)

**Supplementary Table 1: Hypothesis-Driven Genetic Variant Associations with Tau-PET**

| Gene Symbol | Variant | Citation | P-Value |
| --- | --- | --- | --- |
| *CR1* | rs6656401 | (Lambert *et al.*, 2013) | 0.38 |
| *CR1* | rs4844610 | (Kunkle *et al.*, 2019) | 0.59 |
| *BIN1* | rs6733839 | (Lambert *et al.*, 2013) | 0.15 |
| *INPP5D* | rs10933431 | (Kunkle *et al.*, 2019) | 0.11 |
| *INPP5D* | rs35349669 | (Lambert *et al.*, 2013) | 0.31 |
| *MEF2C* | rs190982 | (Lambert *et al.*, 2013) | 0.87 |
| *HLA-DRB1* | rs9271058 | (Kunkle *et al.*, 2019) | N/A^1^ |
| *HLA-DRB1* | rs9271192 | (Lambert *et al.*, 2013) | 0.28 |
| *OARD1* | rs114812713 | (Kunkle *et al.*, 2019) | 0.17 |
| *TREM2* | rs75932628 | (Kunkle *et al.*, 2019) | 0.61 |
| *TREML2* | rs9381040 | (Kunkle *et al.*, 2019) | 0.39 |
| *CD2AP* | rs9473117 | (Kunkle *et al.*, 2019) | 0.38 |
| *CD2AP* | rs10948363 | (Lambert *et al.*, 2013) | 0.27 |
| *NME8* | rs2718058 | (Lambert *et al.*, 2013) | 0.98 |
| *ZCWPW1* | rs1476679 | (Lambert *et al.*, 2013) | 0.91 |
| *NYAP1* | rs12539172 | (Kunkle *et al.*, 2019) | 0.81 |
| *EPHA1* | rs10808026 | (Kunkle *et al.*, 2019) | 0.83 |
| *EPHA1* | rs11771145 | (Lambert *et al.*, 2013) | 0.89 |
| *PTK2B* | rs28834970 | (Lambert *et al.*, 2013) | 0.84 |
| *PTK2B* | rs73223431 | (Kunkle *et al.*, 2019) | 0.22 |
| *CLU* | rs9331896 | (Lambert *et al.*, 2013) | 0.43 |
| *ECHDC3* | rs7920721 | (Kunkle *et al.*, 2019) | 0.91 |
| *SPI1* | rs3740688 | (Kunkle *et al.*, 2019) | 0.41 |
| *CELF1* | rs10838725 | (Lambert *et al.*, 2013) | 0.85 |
| *MS4A6A* | rs983392 | (Lambert *et al.*, 2013) | 0.96 |
| *MS4A (cluster)* | rs7933202 | (Kunkle *et al.*, 2019) | 0.07 |
| *PICALM* | rs10792832 | (Lambert *et al.*, 2013) | 0.06 |
| *PICALM* | rs3851179 | (Kunkle *et al.*, 2019) | 0.44 |
| *SORL1* | rs11218343 | (Lambert *et al.*, 2013) | 0.83 |
| *FERMT2* | rs17125944 | (Lambert *et al.*, 2013) | 0.50 |
| *SLC24A4* | rs10498633 | (Lambert *et al.*, 2013) | 0.64 |
| *SLC24A4* | rs12881735 | (Kunkle *et al.*, 2019) | 0.52 |
| *ADAM10* | rs593742 | (Kunkle *et al.*, 2019) | 0.56 |
| *IQCK* | rs7185636 | (Kunkle *et al.*, 2019) | 0.44 |
| *WWOX* | rs62039712 | (Kunkle *et al.*, 2019) | 0.96 |
| *ACE* | rs138190086 | (Lambert *et al.*, 2013) | 0.34 |
| *DSG2* | rs8093731 | (Lambert *et al.*, 2013) | 0.77 |
| *ABCA7* | rs3752246 | (Kunkle *et al.*, 2019) | 0.77 |
| *ABCA7* | rs4147929 | (Lambert *et al.*, 2013) | 0.07 |
| *APOE* | rs429358 | (Corder *et al.*, 1993) | 0.91 |
| *APOE* | rs7412 | (Corder *et al.*, 1994) | 0.50 |
| *CD33* | rs3865444 | (Lambert *et al.*, 2013) | 0.13 |
| *CASS4* | rs6024870 | (Kunkle *et al.*, 2019) | 0.23 |
| *CASS4* | rs7274581 | (Lambert *et al.*, 2013) | 0.47 |
| *ADAMTS1* | rs2830500 | (Kunkle *et al.*, 2019) | 0.28 |
| *MAPT* | rs4792891  rs9303523  rs1864325  rs1467967  rs117166686  rs2316776  rs62058963  rs12947764  rs242557  rs242559  rs16940758  rs117559252  rs116204525  rs1800547  rs3785883  rs1981997  rs3785885  rs63750072  rs17651549  rs2435212  rs2435214  rs2435200  rs117199550  rs117455029  rs117499775  rs8070723  rs2435203  rs118109282  rs9468  rs1052587  rs16940802 | (Kauwe *et al.*, 2008; Cruchaga *et al.*, 2010; Allen *et al.*, 2014; Peterson *et al.*, 2014; Desikan *et al.*, 2015); others | 0.03  0.03  0.31  0.04  0.46  0.62  0.33  0.25  0.40  0.38  0.30  0.73  0.91  0.27  0.05  0.27  0.19  0.18  0.27  0.51  0.66  0.62  0.11  0.23  0.91  0.27  0.55  0.84  0.29  0.27  0.33 |

^1^SNP not available for analysis due to not passing quality control with imputation

**Supplementary Figure 1: Quantile-Quantile (Q-Q) Plot for the Genome-Wide Association Study of Tau-PET**

Observed -log_10_ p-values (y-axis) were plotted (red dots) against those expected under the null hypothesis (x-axis). No substantial deviation from the black identity line was observed in the bulk of the distribution and the genomic inflation factor (λ) was 1.00, indicating no evidence of spurious inflation of association test statistics due to population stratification or other confounding factors.


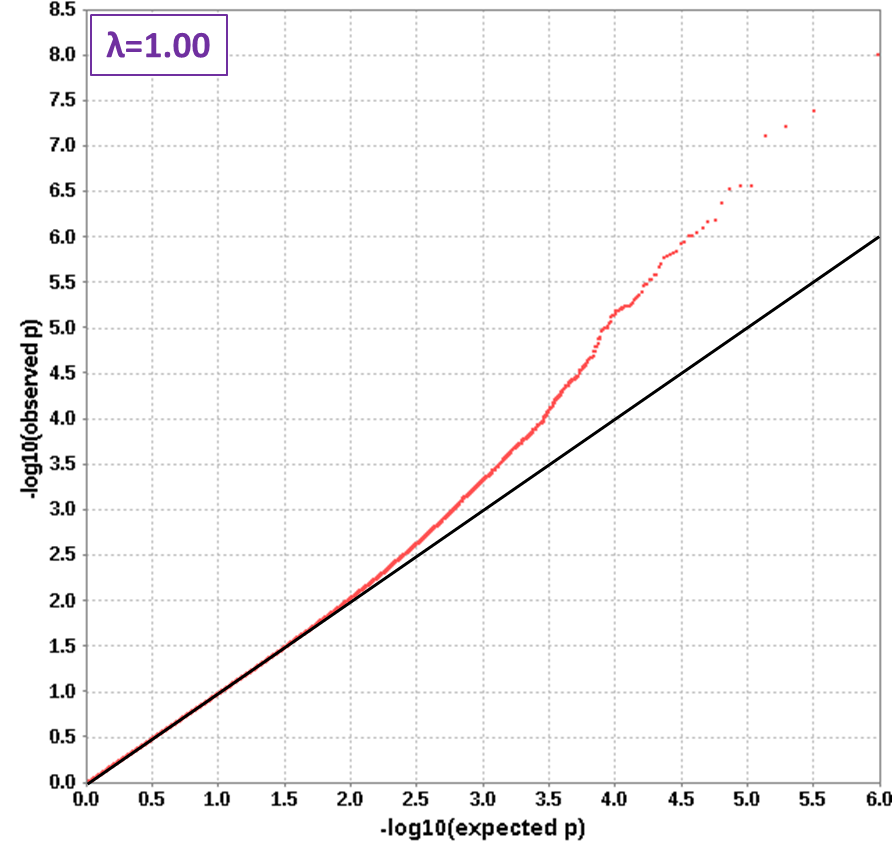


**Supplementary Table 2: Top SNPs from the Genome-Wide Association Study of Tau-PET**

| CHR | SNP | Gene (Nearest) | Position (GRCh37) | Minor Allele | MAF | Beta (std.) | P-Value |
| --- | --- | --- | --- | --- | --- | --- | --- |
| 5 | rs76752255 | *PPP2R2B* | 146135209 | C | 0.015 | 0.20 | 9.91 x 10^-9^ |
| 7 | rs117402302 | Intergenic (*IGF2BP3*) | 23520939 | A | 0.015 | 0.19 | 4.00 x 10^-8^ |
| 2 | rs57978791 | *MPP4* | 202548604 | T | 0.022 | 0.19 | 5.99 x 10^-8^ |
| 1 | rs115862481 | Intergenic (*ST6GALNAC3*) | 76496816 | T | 0.011 | 0.18 | 7.71 x 10^-8^ |
| 3 | rs80226492 | *COX17* | 119389468 | T | 0.026 | 0.18 | 2.66 x 10^-7^ |
| 3 | rs115786849 | *COX17* | 119392243 | A | 0.026 | 0.18 | 2.66 x 10^-7^ |
| 2 | rs73053643 | *MPP4* | 202554073 | C | 0.021 | 0.18 | 2.89 x 10^-7^ |
| 4 | rs11722856 | *JAKMIP1* | 6126144 | A | 0.016 | 0.17 | 4.25 x 10^-7^ |
| 1 | rs74138196 | Intergenic | 188856696 | C | 0.019 | 0.17 | 6.52 x 10^-7^ |
| 8 | rs117603268 | *DLGAP2* | 821593 | G | 0.025 | 0.17 | 6.81 x 10^-7^ |
| 17 | rs35994633 | *PLXDC1* | 37297823 | A | 0.042 | 0.17 | 7.92 x 10^-7^ |
| 21 | rs74688837 | *MX2* | 42767785 | T | 0.032 | 0.17 | 8.99 x 10^-7^ |
| 3 | rs17033066 | *ATP2B2* | 10591192 | T | 0.013 | 0.17 | 9.63 x 10^-7^ |
| 13 | rs9580043 | *LOC105370105* | 21834062 | G | 0.015 | 0.17 | 9.67 x 10^-7^ |

Abbreviations: CHR = chromosome, MAF = minor allele frequency in study dataset

**Supplementary Figure 2:** ***PPP2R2B* rs76752255 and *IGF2BP3* rs117402302**

Boxplots are displayed for the tau-PET meta-region of interest standardized uptake value ratio (SUVR) for all subjects, unadjusted for covariates, and based on genotype for *PPP2R2B* rs76752255 (top) and *IGF2BP3* rs117402302 (bottom). The blue box outlines the interquartile range, with median value denoted by the line inside the box and with values outside the interquartile range denoted by dots.


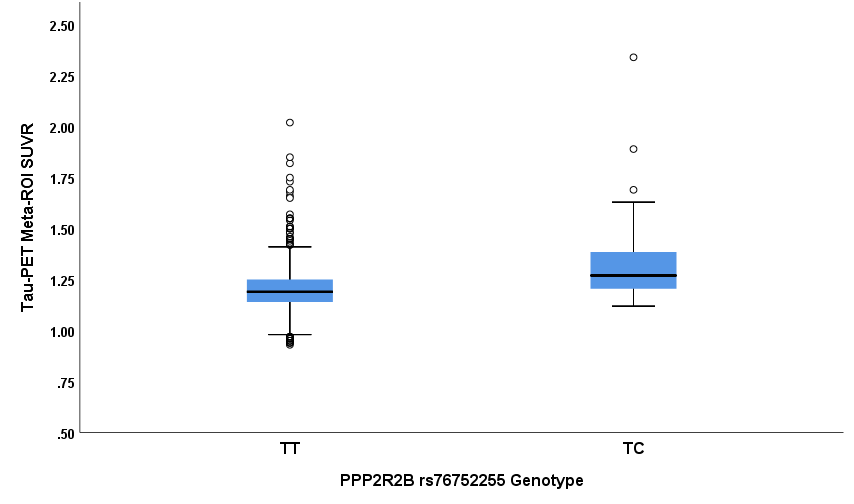


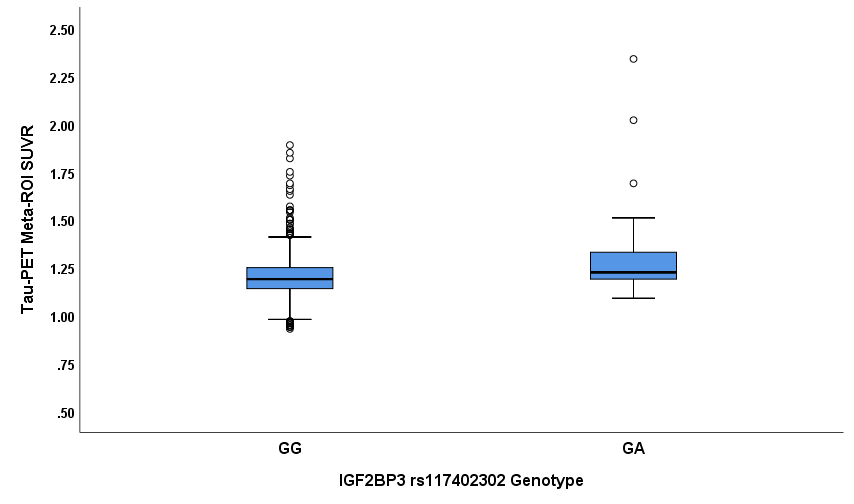


**Supplementary Table 3: Characteristics of the Sample by *PPP2R2B* rs76752255 Genotype**

| Characteristic | All (N=754) | TT (N=731) | TC (N=23) | P-Value |
| --- | --- | --- | --- | --- |
| Age (years) | 71.9 (10.4) | 71.8 (10.4) | 76.3 (9.5) | 0.04 |
| Sex (male) | 412 (55%) | 401 (55%) | 11 (48%) | 0.48 |
| Education (years) | 14.8 (2.6) | 14.8 (2.6) | 15.3 (2.2) | 0.18 |
| CMC | 2.0 (1.6) | 2.0 (1.6) | 1.9 (1.6) | 0.21 |
| Charlson Index | 2.9 (2.9) | 2.9 (2.8) | 4.7 (4.0) | 0.02 |
| Number of Active Medications | 8.0 (4.9) | 8.0 (4.9) | 7.9 (4.1) | 0.42 |
| Mini Mental Status Exam Score | 28.3 (1.7) | 28.4 (1.7) | 27.6 (2.5) | 0.15 |
| Clinical Dementia Rating – Sum of Boxes | 0.2 (0.9) | 0.2 (0.8) | 1.3 (2.9) | <0.001 |
| *APOE* ɛ4 Positivity | 218 (29%) | 212 (29%) | 6 (26%) | 0.71 |
| Global Cortical PiB SUVR | 1.60 (0.44) | 1.60 (0.43) | 1.75 (0.57) | 0.54 |

Data presented as mean (standard deviation) for continuous variables and number (percentage) for categorical variables. P-values for group differences (rs76752255-TT vs. rs76752255-TC) were obtained from an ANCOVA model for continuous variables and logistic regression for categorical variables, with adjustment for age and sex where appropriate. Abbreviations: CMC = index score of cardiovascular and metabolic conditions (range 0-7); SUVR = standardized uptake value ratio

**Supplementary Table 4: Characteristics of the Sample by *IGF2BP3* rs117402302 Genotype**

| Characteristic | All (N=753) | GG (N=731) | GA (N=22) | P-Value |
| --- | --- | --- | --- | --- |
| Age (years) | 71.9 (10.4) | 71.9 (10.4) | 71.2 (10.2) | 0.76 |
| Sex (male) | 412 (55%) | 399 (55%) | 13 (59%) | 0.67 |
| Education (years) | 14.8 (2.6) | 14.8 (2.6) | 14.9 (2.6) | 0.90 |
| CMC | 2.0 (1.6) | 2.0 (1.6) | 2.1 (1.6) | 0.58 |
| Charlson Index | 2.9 (2.9) | 3.0 (2.9) | 2.7 (2.6) | 0.78 |
| Number of Active Medications | 8.0 (4.9) | 8.0 (4.9) | 8.1 (4.8) | 0.79 |
| Mini Mental Status Exam Score | 28.3 (1.7) | 28.3 (1.7) | 28.3 (1.7) | 0.86 |
| Clinical Dementia Rating – Sum of Boxes | 0.2 (0.9) | 0.2 (0.8) | 1.0 (2.7) | <0.001 |
| *APOE* ɛ4 Positivity | 217 (29%) | 212 (29%) | 5 (23%) | 0.52 |
| Global Cortical PiB SUVR | 1.60 (0.44) | 1.59 (0.43) | 1.85 (0.57) | <0.001 |

Data presented as mean (standard deviation) for continuous variables and number (percentage) for categorical variables. P-values for group differences (rs117402302-GG vs. rs117402302-GA) were obtained from an ANCOVA model for continuous variables and logistic regression for categorical variables, with adjustment for age and sex where appropriate. Abbreviations: CMC = index score of cardiovascular and metabolic conditions (range 0-7); SUVR = standardized uptake value ratio

**Supplementary Table 5: Peak Regional Clusters of Voxel-Wise Association of *PPP2R2B* rs76752255 and *IGF2BP3* rs117402302 with Higher Tau-PET Burden**

|  | **Region** | **Cluster size (mm^3^)** | **Cluster FWE*_p_*** | **Peak FWE*_p_*** | **Z-Value** | **Peak Coordinates (x,y,z)** |
| --- | --- | --- | --- | --- | --- | --- |
| *PPP2R2B* | Left Middle Temporal | 459140 | <0.001 | <0.001 | 7.31 | (-58,-38,-10) |
|  | Left Superior Orbital Frontal | 811 | <0.001 | <0.001 | 5.65 | (-14,12,-16) |
|  | Left Middle Frontal | 623 | <0.001 | <0.001 | 5.59 | (-24,0,40) |
|  | Right Supplementary Motor Area | 181 | 0.003 | <0.001 | 5.48 | (8,6,52) |
| *IGF2BP3* | Right Inferior Temporal | 14433 | <0.001 | <0.001 | 6.19 | (51,-6,-32) |
|  | Left Middle Frontal | 503 | <0.001 | <0.001 | 5.69 | (-34,4,27) |
|  | Left Middle Temporal | 3937 | <0.001 | 0.001 | 5.44 | (-60,-36,-12) |
|  | Right Precuneus | 1520 | <0.001 | 0.001 | 5.41 | (6,-56,27) |
|  | Right Superior Frontal | 328 | 0.001 | 0.002 | 5.20 | (22,12,39) |
|  | Left Inferior Temporal | 297 | 0.001 | 0.002 | 5.13 | (-36,-9,-36) |
|  | Left Inferior Parietal | 184 | 0.003 | 0.003 | 5.09 | (-50,-48,32) |
|  | Right Middle Frontal | 198 | 0.003 | 0.009 | 4.85 | (34,14,34) |

**Supplementary Figure 3: Voxel-wise Whole Brain Analyses for the Association of the *APOE* ɛ4 Allele with Tau-PET**

Glass brain surface renderings from whole-brain voxel-wise analyses are displayed. Intensity scales are shown below, with brighter colors on the primary red-orange-yellow scale indicating progressively stronger association signals. In comparison to non-carriers, *APOE* ɛ4 carriers displayed higher tau in focal areas of the left (left panel) and right (right panel) medial temporal region when covarying for age, sex, and genetic principal components. These modest results for *APOE* are in contrast to the robust voxel-wise findings for the *PPP2R2B* and *IGF2BP3* variants identified through GWAS (manuscript Figure 4).


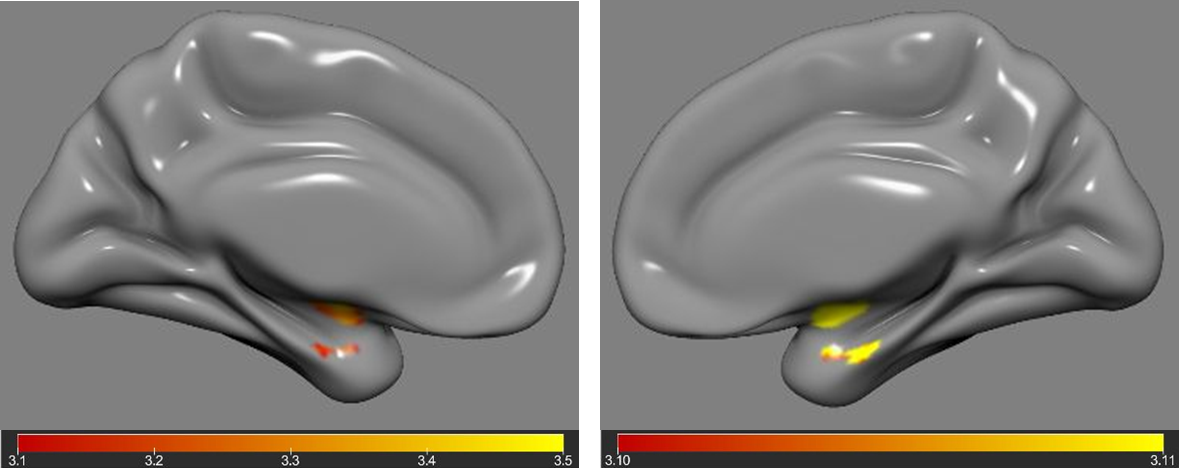


**References**

Allen M, Kachadoorian M, Quicksall Z, Zou F, Chai HS, Younkin C, et al. Association of MAPT haplotypes with Alzheimer's disease risk and MAPT brain gene expression levels. Alzheimer's research & therapy. 2014;6(4):39.

Corder EH, Saunders AM, Risch NJ, Strittmatter WJ, Schmechel DE, Gaskell PC, Jr., et al. Protective effect of apolipoprotein E type 2 allele for late onset Alzheimer disease. Nat Genet. 1994;7(2):180-4.

Corder EH, Saunders AM, Strittmatter WJ, Schmechel DE, Gaskell PC, Small GW, et al. Gene dose of apolipoprotein E type 4 allele and the risk of Alzheimer's disease in late onset families. Science. 1993;261(5123):921-3.

Cruchaga C, Kauwe JS, Mayo K, Spiegel N, Bertelsen S, Nowotny P, et al. SNPs associated with cerebrospinal fluid phospho-tau levels influence rate of decline in Alzheimer's disease. PLoS genetics. 2010;6(9):e1001101.

Desikan RS, Schork AJ, Wang Y, Witoelar A, Sharma M, McEvoy LK, et al. Genetic overlap between Alzheimer's disease and Parkinson's disease at the MAPT locus. Mol Psychiatry. 2015;20(12):1588-95.

Kauwe JS, Cruchaga C, Mayo K, Fenoglio C, Bertelsen S, Nowotny P, et al. Variation in MAPT is associated with cerebrospinal fluid tau levels in the presence of amyloid-beta deposition. Proc Natl Acad Sci U S A. 2008;105(23):8050-4.

Kunkle BW, Grenier-Boley B, Sims R, Bis JC, Damotte V, Naj AC, et al. Genetic meta-analysis of diagnosed Alzheimer's disease identifies new risk loci and implicates Abeta, tau, immunity and lipid processing. Nat Genet. 2019;51(3):414-30.

Lambert JC, Ibrahim-Verbaas CA, Harold D, Naj AC, Sims R, Bellenguez C, et al. Meta-analysis of 74,046 individuals identifies 11 new susceptibility loci for Alzheimer's disease. Nat Genet. 2013;45(12):1452-8.

Peterson D, Munger C, Crowley J, Corcoran C, Cruchaga C, Goate AM, et al. Variants in PPP3R1 and MAPT are associated with more rapid functional decline in Alzheimer's disease: the Cache County Dementia Progression Study. Alzheimers Dement. 2014;10(3):366-71.
